# Excitation landscape of the CP43 photosynthetic antenna complex from multiscale simulations†

**DOI:** 10.1039/d3sc06714a

**Published:** 2024-04-09

**Authors:** Sinjini Bhattacharjee, Srilatha Arra, Isabella Daidone, Dimitrios A. Pantazis

**Affiliations:** a Max-Planck-Institut für Kohlenforschung Kaiser-Wilhelm-Platz 1 45470 Mülheim an der Ruhr Germany dimitrios.pantazis@kofo.mpg.de; b Department of Physical and Chemical Sciences, University of L'Aquila Via Vetoio (Coppito 1) 67010 L'Aquila Italy isabella.daidone@univaq.it

## Abstract

Photosystem II (PSII), the principal enzyme of oxygenic photosynthesis, contains two integral light harvesting proteins (CP43 and CP47) that bind chlorophylls and carotenoids. The two intrinsic antennae play crucial roles in excitation energy transfer and photoprotection. CP43 interacts most closely with the reaction center of PSII, specifically with the branch of the reaction center (D1) that is responsible for primary charge separation and electron transfer. Deciphering the function of CP43 requires detailed atomic-level insights into the properties of the embedded pigments. To advance this goal, we employ a range of multiscale computational approaches to determine the site energies and excitonic profile of CP43 chlorophylls, using large all-atom models of a membrane-bound PSII monomer. In addition to time-dependent density functional theory (TD-DFT) used in the context of a quantum-mechanics/molecular-mechanics setup (QM/MM), we present a thorough analysis using the perturbed matrix method (PMM), which enables us to utilize information from long-timescale molecular dynamics simulations of native PSII-complexed CP43. The excited state energetics and excitonic couplings have both similarities and differences compared with previous experimental fits and theoretical calculations. Both static TD-DFT and dynamic PMM results indicate a layered distribution of site energies and reveal specific groups of chlorophylls that have shared contributions to low-energy excitations. Importantly, the contribution to the lowest energy exciton does not arise from the same chlorophylls at each system configuration, but rather changes as a function of conformational dynamics. An unexpected finding is the identification of a low-energy charge-transfer excited state within CP43 that involves a lumenal (C2) and the central (C10) chlorophyll of the complex. The results provide a refined basis for structure-based interpretation of spectroscopic observations and for further deciphering excitation energy transfer in oxygenic photosynthesis.

## Abstract 2

QM/MM simulations and the perturbed matrix method are used to investigate a crucial photosynthetic antenna complex, mapping its global excitonic structure and revealing the presence of a low-lying charge transfer state.

## Graphical abstract

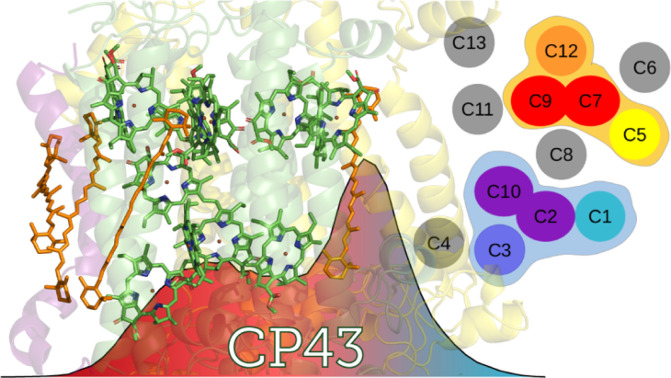

## 1. Introduction

**Fig. 1:**
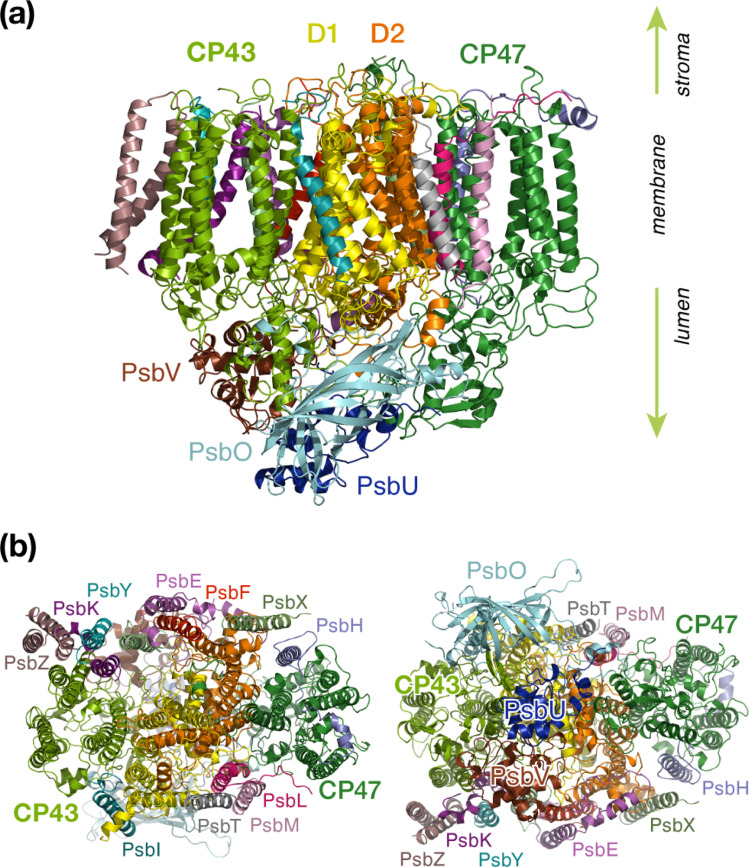
PSII monomer with labeled subunits: (a) side view, (b) stromal and lumenal views.

Oxygenic photosynthesis is a process of fundamental biological and geochemical significance, encompassing light harvesting, charge separation, and water oxidation, centered on Photosystem II (PSII).^1–3^ The cyanobacterial PSII is a dimeric membrane-bound pigment–protein complex comprising 20 protein subunits (17 membrane-intrinsic and 3 extrinsic), along with nearly 100 cofactors in each monomer.^4–6^ The D1 (PsbA) and D2 (PsbD) proteins harbor the reaction center (RC) of PSII, the set of 4 chlorophylls and 2 pheophytins responsible for charge separation that eventually drives H_2_O oxidation to molecular O_2_ by the oxygen-evolving complex,^4,7–11^ coupled to plastoquinone reduction.^12^ Two transmembrane chlorophyll-binding proteins CP43 (PsbC) and CP47 (PsbB), with approximate molecular weights of 43 kDa and 47 kDa respectively, are essential intrinsic core antenna proteins of PSII.^13,14^ They interact closely with the D1 and D2 proteins (Fig. 1) to deliver excitation energy to the RC,^15,16^ working either as light absorbers themselves or facilitating excitation energy transfer (EET) from peripheral light-harvesting complexes.^3,17–25^

**Fig. 2:**
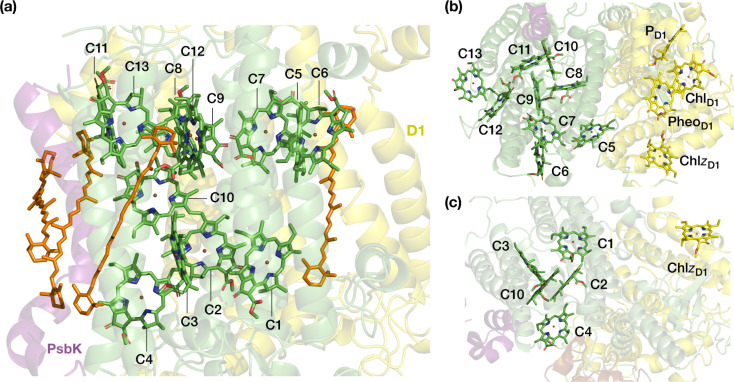
The pigments of the CP43 complex with their labels: (a) side view, indicating also positions of carotenoids; (b) stromal (“top”) view, depicting also proximal pigments of the reaction center that belong to the D1 (PsbA) chain; (c) lumenal (“bottom”) view. Chlorophyll C10 is shown in both panels on the right to aid orientation. C5 is the closest CP43 chlorophyll to D1 pigments Pheo_D1_ and Chl*z*_D1_ (*ca.* 21–22 Å, see ESI† for details).

CP43 is closest to the D1 protein, which hosts the branch of RC pigments that are active in charge separation^26^ and that also accommodates the Mn_4_CaO_*x*_ cluster of the oxygen-evolving complex (OEC), the site of water oxidation. In addition to its role in EET, CP43 plays a pivotal role in maintaining the overall structural integrity of the RC and contributes to the stabilization of the OEC itself by providing a direct manganese-coordinating ligand (Glu354) as well as crucial second-sphere functionality (Arg357). As an essential core antenna complex, CP43 has been the subject of numerous studies that attempted to elucidate its spectroscopic properties and excitonic structure.^13,27–31^ CP43 contains 13 embedded Chl *a* pigments and 4 β-carotenes. Fig. 2 depicts the spatial arrangement of the CP43 chlorophylls and indicates the labeling used in the present work, which follows the numbering recommended by Müh and Zouni.^18^Table 1 describes distinctive characteristics of each chlorophyll in terms of their axial ligation and the hydrogen-bonding interaction at the 13^1^-keto group. Additionally, to facilitate comparisons with previous studies that follow structure-specific numbering of CP43 chlorophylls, Table 1 lists the corresponding numbering of the CP43 chlorophylls in selected PSII crystallographic models. As shown in Fig. 2, the chlorophylls are distributed in three layers, with four Chl molecules present in the lumenal layer (C1–C4), eight in the stromal layer (C5–C9, C11–C13), and one (C10) positioned in the center of the lipid bilayer. This arrangement is reminiscent of the distribution of chlorophylls in the CP47 antenna.^13,32^

**Table 1:** Nomenclature for the CP43 chlorophylls. His_δ_ and His_ε_ denote the binding mode of histidine residues with respect to the N_δ_ and N_ε_ site, respectively. Axial ligation on the opposite and the same side of the phytyl chain are denoted as α and β type ligation. The chlorophylls in the lumenal and stromal domain are denoted as L and S, respectively. All amino acid residues are from CP43 (PsbC subunit) unless otherwise indicated

Site	Axial	Keto H-bond	Location	2AXT^33^	3BZ1 (ref. 34)	3WU2-A^35^	3WU2-B	4IL6 (ref. 36)
C1	His_ε_237(α)	H_2_O	L	33	474	501	902	501
C2	His_ε_430(α)	Tyr297	L	34	475	502	903	502
C3	His_ε_118(α)	—	L	35	476	503	904	503
C4	H_2_O(α)	H_2_O, LMG-519	L	37	477	504	905	504
C5	His_ε_441(α)	H_2_O, Arg449	S (close to D1)	41	478	505	906	505
C6	His_ε_251(α)	—	S	42	479	506	907	506
C7	H_2_O(α)	His_δ_164	S	43	480	507	908	507
C8	His_ε_444(α)	—	S (close to D1)	44	481	508	909	508
C9	His_ε_53(β)	Ser275	S	45	482	509	910	509
C10	His_ε_56(α)	—	Center (close to PsbK)	46	483	510	911	510
C11	Asn39(α)	H_2_O, Arg41	S (close to PsbK)	47	484	511	912	511
C12	His_ε_164(α)	H_2_O	S	48	485	512	913	512
C13	His_ε_132(α)	Tyr131, LMT 102	S	49	486	513	914	513

Spectroscopic characterizations of intrinsic antenna complexes CP43 and CP47 are often performed on samples extracted from PSII. In the case of isolated CP43 samples thus only 13 chlorophylls contribute to the spectra, facilitating analysis and fitting of the spectra to some extent. However, isolated samples depart from the native structure and possibly lack the structural integrity of the PSII-complexed system, potentially introducing inconsistencies in the resulting data sets. Additionally, it is possible that isolated preparations or certain treatments may result in deformation or even loss of one or more chlorophylls, as suggested for CP47.^32^

The CP43 core antenna in PSII is anticipated to possess two quasi-degenerate low-energy “trap” states,^31,37^ inferred from hole-burning (HB) studies,^38^ other spectroscopic investigations^39,40^ and structure-based calculations.^27–29^ However, the exact assignment of these low energy excitonic states remains a topic of active debate in literature. Shibata *et al.* reported the presence of two red-shifted pigment domains within the PSII core complex emitting at 685 nm and 695 nm,^16^ the former assigned to CP43. Previously, Hughes *et al.* claimed that both states are localized on one chlorophyll, but one is excitonically coupled to other states.^37^ On the other hand, Raszewski, Renger and Müh have associated one CP43 trap state with a localized exciton in the lumenal layer whereas the other trap state was concluded to be on a delocalized domain in the stromal layer.^29,41^

The kinetics of EET are also debated, with different groups arriving at significantly different values for the transfer times from the CP43/CP47 antennae to the RC compared to the rate of primary charge separation (CS) at the RC.^15,16,42,43^ The conclusions depend on the distinct assumptions and theoretical models employed. For example, Holzwarth and co-workers reported timescales of a few ps for EET to the RC based on transient absorption and fluorescence kinetics,^44,45^ whereas Renger arrived at estimates approximately an order of magnitude slower for the forward and half of that for the backward EET process.^41,46^ This also led to alternate EET mechanisms being proposed, namely the “exciton-radical pair equilibrium” (ERPE) model^30,44^ and the “transfer-to-trap limited” (TTTL) model.^45,47^ It has also been suggested that CS and EET may occur on the same timescale.^14^ More recently, Yang *et al.* investigated the EET dynamics of the PSII core complex using two-dimensional electronic-vibrational (2DEV) spectroscopy^19^ and suggested that C5 (current labeling) in CP43 and the peripheral D1 chlorophyll known as Chl*z*_D1_ likely form the pathway for energy transfer from CP43 to the RC. The results were consistent with the TTTL model in the sense that EET from CP43 to Chl*z*_D1_ was found to be faster than subsequent EET to other D1 pigments, a step which thus constitutes the kinetic bottleneck.

Spectroscopic studies towards determining possible EET pathways in photosynthetic light harvesting complexes (LHCs) still face two main challenges.^17,48,49^ First, the closely spaced pigments exhibit significant excitonic couplings rendering it impossible to make a direct correlation between the absorption bands and individual pigments. Second, the highly congested excitonic manifold makes it non-trivial to assign site energies to specific Chl molecules. That is why theoretical approaches that complement experimental data (*e.g.* spectral densities, absorption and fluorescence spectra) by simulating the structures and estimating the site energies of pigment–protein complexes have long played an important role in the study of antenna complexes.^25,28,29,50–58^ Past investigations employed quantum mechanics/molecular mechanics (QM/MM) and molecular dynamics (MD) simulations based on predetermined potential energy surfaces (PES) derived from DFT,^59^ or “on-the-fly” PES obtained from the semi-empirical DFTB approach.^60^ However, it remains challenging even with approximate QM methods to perform state of the art simulations on groups of chlorophyll pigments in systems such as CP43 and CP47 while simultaneously taking fully into account the short- and long-range effects of the protein matrix. Furthermore, even in existing theoretical studies there have been discrepancies regarding the relative ordering of site energies and identity of the low energy trap states.^38,39^ In two investigations, Müh, Renger and co-workers identified three red chlorophylls in the isolated CP43 protein.^28,29^ The excitonic couplings and local transition energies of chlorophylls were computed using Poisson–Boltzmann (PB) electrostatics in both cases, albeit the latter study was based on the high-resolution crystal structure of PSII.^28,29^ Saito *et al.* evaluated EET coupling between Chls in the PSII monomer based on the QM/MM diabatization scheme.^61^ In a more recent computational study, Sarngadharan *et al.* determined the site energies and excitonic couplings of CP43 chlorophylls^27^ using time-dependent long-range corrected density functional tight binding approach (TD-LC-DFTB) and QM/MM MD trajectories. All the above theoretical studies seemed to have reached a consensus that the red-most chlorophylls in CP43 are delocalized and likely belong to two separate “domains” of the transmembrane region. It is also important to note that all or most of the theoretical studies are based on the isolated CP43 protein, without the remaining PSII matrix. Moreover, axial ligation to the central Mg atom, pigment–protein interactions (*e.g.*, H-bonding to the keto group), and pigment–pigment interactions (*e.g.* in Chl dimers) are known to directly influence excited state properties of chlorophylls,^32,62,63^ it is therefore crucial to treat these interactions at the QM level along with the chromophore, an aspect which has been overlooked in several past studies.

Here we utilize a comprehensive large-scale QM/MM approach employing range-separated time-dependent density functional theory (TD-DFT) to investigate the low-energy excited states for all CP43 chlorophylls. The calculations explicitly account for interactions of the pigments with the complete membrane-embedded all-atom PSII monomer. Beyond individual pigments, we apply the same methods on pigment dimers in order to study coupled pairs and to investigate the presence of possible charge-transfer (CT) states.^62,64,65^ Crucially, our work incorporates the perturbed matrix method (PMM)^66–69^ that enables us to leverage the full information from long-timescale molecular dynamics simulations for the extraction of site energies and excitonic couplings. In addition to a new set of site energies and excitonic couplings, the results identify a previously unknown low-lying state with significant charge transfer character among CP43 chlorophylls, which may have important implications for the functional role of the protein. Combined with a refined analysis of the contributions of the different monomeric and dimeric pigment groups to the first exciton, the present results provide a detailed map of static and dynamic properties of the CP43 pigments and contributes to the improved understanding of this essential photosynthetic antenna.

## 2. Methodology

### 2.1. QM/MM geometries

The QM/MM computations on the CP43 chlorophylls are performed based on an equilibrated PSII-lipid bilayer model of the 3WU2 crystal structure (Fig. S1†).^35,62,63^ The detailed protocols for equilibration and production dynamics are discussed in the ESI.† We consider the entire PSII monomer and a water layer (7 Å around the protein), including all the waters present in the protein cavity and various channels. The final system used in the QM/MM calculations consisted of 76 035 atoms. All QM/MM calculations were performed using the multiscale module of the ORCA 5.0 suite,^70,71^ employing the electrostatic embedding technique. The hydrogen link atom approach was employed to cut through C–C covalent bonds and the charge-shift (CS) scheme was used to avoid overpolarization of the QM region. For each chlorophyll, the chlorin macrocycles along with the axial ligand to Mg^2+^ and the side chain of residues H-bonded to the keto group at the 13^1^-carbon position (ring E) are included in the QM region. The phytyl chains were included in the QM region up to C^17^ (truncated as a methyl group) and the rest of the chain was treated in the MM region. For geometry optimizations, the complete system was further subdivided into two parts: active and static. The active region consists of atoms within the QM and MM regions that remain free to move during optimization, whereas the remaining MM atoms are fixed and only contribute to the electrostatics. Complete amino acid residues and waters within 10 Å from the center of each chlorin ring (taken as the Mg^2+^) were considered in the active region. For the chlorophyll pairs, we performed a constrained optimization where the QM/MM optimized geometries of individual pigments were combined and kept fixed while only the MM active region was relaxed. The Perdew–Burke–Ernzerhof (PBE) functional^72,73^ was used to optimize the QM/MM geometries using the def2-TZVP basis set^74^ and D3(BJ) dispersion corrections^75,76^ throughout. The resolution of identity approximation (RI)^77^ was used to speed up the calculation of Coulomb integrals with the corresponding auxiliary basis set (def2/J).^78^

### 2.2. Calculation of excited states

Vertical excitation energies are computed on the optimized ground state geometries using full TD-DFT without the Tamm–Dancoff approximation. All calculations were performed using the range separated ωB97X-D3(BJ) functional (modified version of ωB97X-V^79^ with D3BJ correction) along with def2-TZVP basis sets. This long-range-corrected functional has a fixed exact (Hartree–Fock) exchange of 16.7% (short-range) that increases to 100% at long range with a range-separation parameter of 0.30 bohr^−1^. The first 8 excited states (roots) were computed, thus covering the entire Q-band range and further low-lying excited states for individual chlorophylls as well as for dimers.^26,63,80^ The electrostatic effects of the protein environment were included through MM point charges. The RIJCOSX approximation^81^ and the corresponding auxiliary basis sets were used throughout. VeryTightSCF convergence criteria were applied, along with dense integration grids (DefGrid2). The vertical excitation energies (VEE) and transition dipole moments (to be used in subsequent PMM calculations) for the ground and first two excited states were calculated for all the QM regions specified above. The nature of the excited states was characterized based on Natural Transition Orbitals (NTOs)^82^ using the orca_plot module.

### 2.3. Perturbed matrix method (PMM) calculations

The Perturbed Matrix Method (PMM) shares conceptual similarities with many QM/MM approaches.^83–86^ In this methodology, the system is partitioned into two distinct entities: the Quantum Center (QC), encompassing the specific portion treated at the quantum level, and the remaining component, referred to as “the environment”, described as a semiclassical perturbation acting upon the QC. Differently from conventional QM/MM schemes, PMM employs long timescale molecular dynamics (MD) simulations for the entire system, encompassing both the QC and the environment, all governed by the same Hamiltonian—that is, the same classical force field. In the MD-PMM framework, the unperturbed quantum properties of the isolated QC are first calculated at the QM level on the QC geometry optimized at the QM/MM level (see below). Following this, for each configuration of the simulated system (*i.e.*, for each frame in the classical molecular dynamics simulation), the electrostatic effect of the instantaneous atomistic configuration of the environment is included as a perturbing term within the QC Hamiltonian operator. The electronic Hamiltonian operator *Ĥ* of the QC embedded in the perturbing environment can be thus expressed as follows:

1 *Ĥ* = *Ĥ*^0^ + *V̂*

where *Ĥ*^0^ is the QC unperturbed electronic Hamiltonian (*i.e.*, as obtained considering the isolated QC) and *V̂* is the perturbation operator. The perturbation operator *V̂* can be derived using a multipolar expansion centered on the QC's center of mass:

2 *V̂* = *q*_T_*V* − **E**·***μ̂***

<svg xmlns="http://www.w3.org/2000/svg" version="1.0" width="13.000000pt" height="16.000000pt" viewBox="0 0 13.000000 16.000000" preserveAspectRatio="xMidYMid meet"><metadata>
Created by potrace 1.16, written by Peter Selinger 2001-2019
</metadata><g transform="translate(1.000000,15.000000) scale(0.012500,-0.012500)" fill="currentColor" stroke="none"><path d="M560 1080 l0 -40 -40 0 -40 0 0 -40 0 -40 -40 0 -40 0 0 -40 0 -40 40 0 40 0 0 40 0 40 40 0 40 0 0 40 0 40 40 0 40 0 0 -40 0 -40 40 0 40 0 0 -40 0 -40 40 0 40 0 0 40 0 40 -40 0 -40 0 0 40 0 40 -40 0 -40 0 0 40 0 40 -40 0 -40 0 0 -40z M320 720 l0 -80 -40 0 -40 0 0 -120 0 -120 -40 0 -40 0 0 -120 0 -120 -40 0 -40 0 0 -80 0 -80 80 0 80 0 0 120 0 120 80 0 80 0 0 40 0 40 40 0 40 0 0 -40 0 -40 120 0 120 0 0 40 0 40 40 0 40 0 0 40 0 40 -40 0 -40 0 0 120 0 120 40 0 40 0 0 80 0 80 -80 0 -80 0 0 -80 0 -80 -40 0 -40 0 0 -80 0 -80 -40 0 -40 0 0 -40 0 -40 -40 0 -40 0 0 120 0 120 40 0 40 0 0 80 0 80 -80 0 -80 0 0 -80z m80 -360 l0 -40 -40 0 -40 0 0 40 0 40 40 0 40 0 0 -40z m320 0 l0 -40 -40 0 -40 0 0 40 0 40 40 0 40 0 0 -40z"/></g></svg>

***

with *q*_T_ the total QC charge, *V* the electrostatic potential exerted by the perturbing environment, **E** the perturbing electric field and ***μ̂*** the dipole operator. Finally, at each MD frame, the diagonalization of the Hamiltonian matrix provides a set of eigenvectors and eigenvalues representing the perturbed eigenstates and energies of the QC. In the present study we are mainly interested in the site energy, namely the difference between the perturbed energies of the first-excited and ground states of each QC. This physical quantity is hence calculated at each frame of the MD simulation, and the average evaluated in the MD ensemble. The unperturbed properties for each QC are calculated using the corresponding QM/MM optimized geometry, which, thus, remains fixed in the MD-PMM calculations.

In case of the interacting chromophores, *i.e.*, a set of interacting QCs, we may consider the possible excitation coupling occurring among the QCs.^87^ In the present case, the interactions between the electronic excitations localized on each Chl are considered, with the exception of C2–C10 and C7–C9 that were considered as dimers due to their electronic coupling (see above). To this aim, the perturbed Hamiltonian operator for the 11 QCs (9 single chlorophylls and 2 dimers) is considered and, in matrix notation, is expressed as follows:

3 *H̃* = *E*_0_*Ĩ* + Δ*H̃*

where *E*_0_ is the electronic ground-state energy, *Ĩ* is the identity matrix and Δ*H̃* is the excitation matrix, the diagonal elements of which are given by the single chromophores perturbed excitation energies. The non-diagonal elements of the excitation matrix *i.e.*, the excitonic couplings, are obtained by truncating the expansion of the interaction operator of the chromophores at the dipolar term *i.e.*, using the point dipole approximation (PDA). Thus, the electronic coupling between two QCs is treated as a dipole–dipole interaction and the *k*,*k'* interaction operator is given by:4
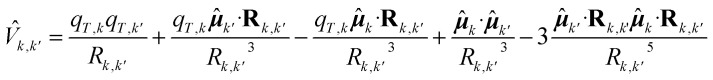
where ***μ̂***_*k*_ is the *k*th chromophore dipole operator and **R**_*k,k*′_ is the *k*′ to *k* chromophore displacement vector defined by the corresponding chromophore centers of mass. It should be noted that this is the general expression for charged QCs. In the present case, all QCs are neutral and thus the first three terms of the equation are zero. The transition dipoles in eqn (4) are the perturbed transition dipoles as obtained by considering each single chromophore embedded in the field produced by the rest of the environment, including the other QCs. Despite the fact that transition charges from the electrostatic potential (TrESP) method are generally considered preferable to PDA for excitonic couplings,^88^ recent studies on the water-soluble chlorophyll-binding protein (WSCP)^89^ and on the Fenna–Matthews–Olson (FMO) complex^59^ showed that the dipole approximation can give an accuracy comparable to TrESP. In the case of the FMO complex, the agreement between PDA and TrESP couplings is quantitative; in the case of the WSCP, PDA couplings were found to be only a few percent larger. In the present study we opted for PDA due to its computational efficiency and because it is already implemented within the PMM framework, facilitating a smoother workflow. Except for the computation of the excitonic states, which were performed using an in-house Fortran code, the basic PMM code is available as an open-source program (PyMM).^90^

Finally, by diagonalizing the excitation matrix, the perturbed excitation energies and eigenstates (*i.e.*, the exciton states) are obtained. In the present case we utilized 3000 frames, corresponding to the final 30 ns of a previously performed 200 ns-long MD trajectory,^64,91^ saved at intervals of 10 ps. This specific time frame was previously employed for the redox potential calculations^65^ and was selected to ensure a reasonably converged root mean square deviation (RMSD) of the entire system with respect to the starting, crystallographic structure. In principle, there is no computational constraint on the number of frames that can be utilized within the MD-PMM framework, as it solely depends on the length of the MD trajectory.

The PMM approach has previously been employed in the study of chlorophylls and pheophytins of the PSII-RC.^65^ This application demonstrated very good agreement with experimentally-derived thermodynamic parameters such as reduction potentials, and with experimental kinetics properties such as rate constants of primary electron transfer within the RC. Here, we further validated the MD-PMM approach by computing the absorption line shape of CP43 at room temperature (see Fig. S2†). This was accomplished using our previously-established procedure, which is outlined in the ESI.† Notably, there is a close match with the experimental line shape. Specifically, the experimental exciton splitting at 293 K (that manifests as a slight shoulder at low energy) is accurately reproduced (12 nm in the computed spectrum *versus* 13 nm in the experimental one). The total bandwidth is slightly overestimated (34 nm *versus* the experimental 24 nm), most probably due to an overestimation of the inhomogeneous broadening provided by the perturbation calculated along the MD trajectory.

## 3. Results and discussion

### 3.1. Chlorophyll site energies

The determination of the low-energy excitation energy profile for CP43 and CP47 core antenna pigments is key in identifying the characteristic pathways of excitation energy transfer (EET) within PSII. Theoretical parameters derived from calculations are frequently employed to estimate excitation energies by fitting them to experimental spectra. Moreover, spectroscopic analysis of CP43 and CP47 antenna proteins is often performed on isolated samples extracted from the larger native PSII core complex. In the case of extracted CP43 thus only 13 chlorophylls contribute to the spectra, which aids in the fitting to some extent, but the data lacks the effect of global PSII electrostatics. It is well established that the PSII matrix largely influences the photochemical properties of chlorophylls through electrostatics, structural distortions, axial ligation and secondary sphere interactions like H-bonding networks. Most importantly, local protein electrostatics are known to be predominantly responsible for fine-tuning the excitation energies of photosynthetic pigments.^62,63,92^ Here we consider all the above factors by employing multiscale modeling techniques within the QM/MM framework to determine the site energies of all CP43 chlorophylls embedded in the “native” PSII system.

**Fig. 3:**
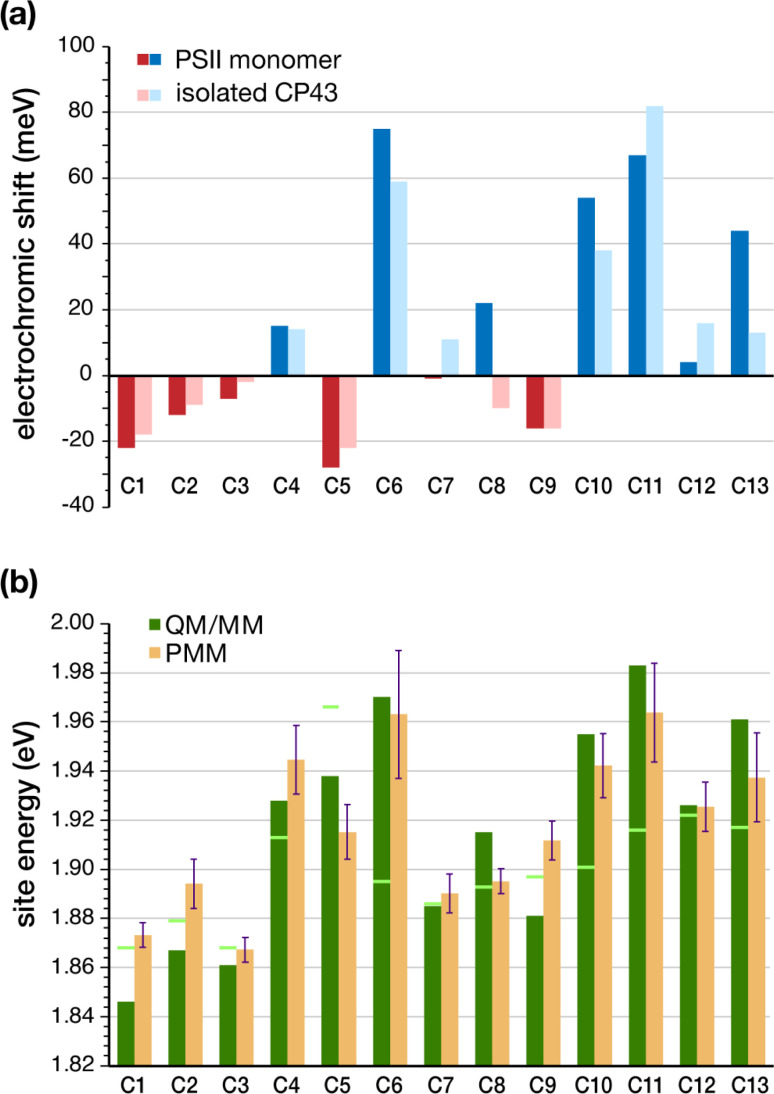
(a) TD-DFT computed electrochromic shifts in site energies (meV) for CP43 chlorophylls compared to gas-phase calculations. The electrochromic shift is defined as S_1_ (embedded) – S_1_ (vacuum) and is shown for both the whole PSII monomer and for the CP43 protein only. (b) Chlorophyll site energies computed with TD-DFT QM/MM on the reference snapshot (gas-phase values indicated as light green lines), compared to the mean site energies obtained from MD-PMM calculations averaged over 3000 frames from the equilibrated portion of the MD simulation.

The geometries of all individual chlorophylls were optimized using DFT and QM/MM as described in the Methodology. Following complete optimizations, we computed the vertical excitation energies of each CP43 chlorophyll *in vacuo* (also referred to commonly as “in the gas phase”) using TD-DFT on the QM/MM optimized geometries. Table 2 provides a comprehensive overview of the vertical excitation energies of the lowest excited state (S_1_ or Q_*y*_), and the protein electrochromic shifts of each CP43 chlorophyll. We find that the *in vacuo* first excited state energies lie within the range of 1.868–1.966 eV (see Table 2). This demonstrates that intrinsic structural features of the pigment itself, like the macrocyclic ring curvature and the nature of axial ligation, play already major roles in differentiating the site energies of each chlorophyll. We subsequently performed excited state calculations on the CP43 protein excluding the rest of the PSII monomer, so that we can compare the effect of having an “isolated” CP43 chain as opposed to a complete PSII monomer (we emphasize that this is not equivalent to simulating an experimentally extracted CP43 antenna because we are not simulating here the conformational state of the latter, a problem that presents a distinct challenge). The results show that the local protein electrostatics already shift the Chl site energies toward the red or blue regime relative to their gas-phase values (Table 2). Overall, the site energies for the isolated CP43 lie in the range of 1.850–1.998 eV. The six chlorophylls C1, C2, C3, C5, C8 and C9 are red-shifted, whereas the remaining C4, C6, C7, C10, C11, C12 and C13 chlorophylls are blue-shifted with respect to the gas-phase excitation energies (Fig. 3). Interestingly, 3 out of the 6 red-shifted chlorophylls are on the lumenal side of the protein (Fig. 2c). These findings are consistent with the stromal *versus* lumenal (membrane-transverse) trends obtained for the pigments in the RC and the CP47 antenna of PSII.^32,62^ Chlorophylls C5 and C1 are the pigments that shift most to the red due to protein matrix electrostatics.

**Table 2:** TD-DFT (ωB97X-D3(BJ)/def2-TZVP) site energies *E* (in eV) of all CP43 chlorophylls and oscillator strengths of the S_1_ (Q_*y*_) transition computed *in vacuo*, with QM/MM on an isolated CP43 protein, and with QM/MM in the complete PSII monomer, compared with the mean site energy obtained by PMM. The geometries in all cases are derived from QM/MM optimizations within the PSII monomer. Shifts are reported in meV with respect to the gas-phase values

Site	TD-DFT gas-phase		TD-DFT|QM/MM isolated CP43			TD-DFT|QM/MM full PSII monomer			PMM	
	*E*	*f*_osc_	*E*	*f*_osc_	Shift	*E*	*f*_osc_	Shift	*E*_mean_	Std. dev.
C1	1.868	0.22	1.850	0.20	−18	1.846	0.19	−22	1.873	0.005
C2	1.879	0.22	1.870	0.20	−9	1.867	0.20	−12	1.894	0.010
C3	1.868	0.22	1.866	0.20	−2	1.861	0.20	−7	1.867	0.005
C4	1.913	0.23	1.927	0.19	14	1.928	0.17	15	1.945	0.014
C5	1.966	0.23	1.944	0.22	−22	1.938	0.25	−28	1.915	0.011
C6	1.895	0.22	1.954	0.21	59	1.970	0.21	75	1.963	0.026
C7	1.886	0.23	1.897	0.20	11	1.885	0.21	−1	1.890	0.008
C8	1.893	0.22	1.883	0.23	−10	1.915	0.23	22	1.895	0.005
C9	1.897	0.23	1.881	0.23	−16	1.881	0.22	−16	1.912	0.008
C10	1.901	0.22	1.939	0.23	38	1.955	0.23	54	1.942	0.013
C11	1.916	0.22	1.998	0.22	82	1.983	0.24	67	1.964	0.020
C12	1.922	0.22	1.938	0.21	16	1.926	0.21	4	1.925	0.010
C13	1.917	0.23	1.930	0.22	13	1.961	0.22	44	1.937	0.018

The identity of the red-shifted chlorophylls remains consistent when we inspect the full results for the complete PSII monomer, but the effect of the protein matrix is now significantly more pronounced. The global PSII protein electrostatics further stabilize the site energies of chlorophylls C1, C2, C3 and C5, with C5 still being the most red-shifted pigment. This result is significant for two reasons. First, C5 (C505 in 3WU2) is located on the periphery of CP43 and is in direct contact with the D1 (PsbA) subunit of PSII. Second, recent two-dimensional electronic-vibrational (2DEV) spectroscopy measurements on the PSII core complex^19^ suggested that chlorophyll C5 along with the peripheral chlorophyll Chl*z*_D1_ likely mediate EET from CP43 to the PSII-RC. The electrochromic shift on chlorophyll C9 (16 meV) remains invariant in the isolated CP43 and in the PSII monomer. This is expected as well because it is deeply buried within the CP43 protein matrix and is the pigment least affected by structural deformation of CP43 upon isolation. Another interesting result is seen for C8, the only pigment to show opposite shifts in the intact PSII monomer compared to isolated CP43. C8 is also located close to the D1 (PsbA) subunit, making it susceptible to conformational changes during extraction from the native PSII. The D1 electrostatics induce an additional blue-shift in the excitation energy of C8. The remaining blue-shifted chlorophylls (C6, C11, C13) are mostly located in the stromal layer of CP43, except for C10 which is in the middle of the transmembrane region. C6 and C13 lie on the periphery of CP43 while C10 and C11 are located close to the PsbK subunit (Fig. 2b). It is suggested that the salt bridge between D2-R233 and CP43-E29 may affect the interaction between C11 and the protein, thus lowering its site energy slightly in the intact PSII compared to extracted samples.^28^

Based on the results on CP43 chlorophylls in the intact PSII monomer, the computed site energies lie in the range of 1.846–1.983 eV while the second excited states (S_2_/Q_*x*_) range from 2.260–2.468 eV (see Table S1†). Moreover, the energy gap between the first two excited states is also seen to vary among the different domains, with the lowest being for C1 and C2 in the lumenal domain, and the highest being for site C8 in the stromal layer close to PsbA (D1). The Q_*x*_–Q_*y*_ energy gap is modulated by both intrinsic (macrocyclic ring curvature) and extrinsic components (axial ligation, H-bonding, protein electrostatics), as also seen for the case of CP47 and RC chlorophylls.^32,92^

It is noted that the differences in site energies of the CP43 chlorophylls do not exclusively arise from protein electrostatics, as significant shifts are observed for the pigments in their QM/MM optimized geometries when the excited state calculations are performed *in vacuo*. We analyzed the contribution of these other effects through excited state calculations on the pigments *in vacuo*, but without the influence of the axial ligation and H-bonding interactions. Differences in the Q_*y*_ energy with that of optimized Chl *a* give the contribution of the QM/MM geometry optimization on the site energies. The results are summarized in Fig. S3.† Our results suggest that protein electrostatics still have the dominant contribution to site energy shifts, but there are significant contributions from the QM/MM geometry optimizations (*i.e.*, intrinsic structural differentiation), especially contributing to the blue-shift of Chls C4 and C9–C13. The axial ligands do not affect the results significantly, but only slightly red-shift the S_1_ state in each case. One important finding is that CP43-R449, which is H-bonded to a water bridging the keto group of C5, individually blue-shifts its site energy to a great extent but overall, the protein matrix overcompensates and tunes it in the opposite direction. These findings resemble past observations on chlorophylls of CP47, which also had a similarly wide distribution of site energies even in the absence of protein electrostatics.^32^ Results on both antennae are in this respect different from results on the PSII RC, where all 4 Chls (Chl_D1_, Chl_D2_, P_D1_ and P_D2_) had essentially the same site energies in the absence of the protein electrostatics.^62^ Given that the influence of the axial ligands is not a decisive factor, it seems that the most plausible explanation for the difference is that the protein matrix allows greater flexibility for the CP43/CP47 chlorophylls, whereas the RC pigments need to be more rigid to allow for efficient electron transfer.

An effective approach for assessing the spectral density of a chromophore within a photosynthetic pigment–protein complex (PPC) involves simulating the fluctuations in site energy resulting from environmental effects.^50,53,93^ However, current limitations arise from the substantial size and the intricacy of these complexes and from the impossibility of simulating a membrane embedded PPC immersed in a water box while applying continuously QM methods for meaningful sampling.^94–96^ Therefore, to account for the effect of conformational changes of the PSII protein environment on the individual site energies, the site energies of individual Chls were also calculated by means of the MD-PMM procedure. The energies are obtained at each time frame of the MD simulation over a specific interval. The corresponding site energy distributions are reported in Fig. S4.† It can be observed that the distributions of the individual pigment molecules differ in their peak positions, widths, and skewness, although all distributions are roughly Gaussian in shape. The corresponding mean site energies are also reported in Table 2 and compared with the results obtained by means of the TD-DFT calculations. Fig. 3b shows that the two sets of site energies have the same overall trend and that also the quantitative agreement is quite high, with maximum absolute deviations (MAD) of 20–30 meV. The maximum deviations are seen for pigments C1, C2, C5, C9 and C13.

In one line of investigation, Raszewski *et al.* associated the “red” chlorophyll (at 685 nm) with C4 (or C504 in 3WU2) in the lumenal layer of CP43.^41^ In subsequent studies relying on high-resolution PSII structures,^28^ the other “trap” state was suggested to be a delocalized excited state involving multiple pigments (C7, C9, and C11) located in the stromal layer. These assignments of trap states were derived largely from theoretical simulations of optical spectra that employed computed excitonic couplings and adapted site energies. A somewhat different perspective was presented by Shibata *et al.*,^16^ who reported the presence of two red-shifted chlorophyll domains within PSII-cc, emitting at 685 nm and 695 nm respectively, based on time-resolved fluorescence spectroscopy. In their study, only the 685 nm band was attributed to either C9 or C7 sites within CP43. A detailed comparison of the chlorophyll site energies obtained from different literature sources is provided in Table S3 of the ESI.† It is important to note that the results presented in the current work are not directly comparable to site energies obtained by approaches that involve fitting to various experimental data sets and different theoretical methods. Nevertheless, we can compare the relative trends in site energies with respect to the chlorophyll with the lowest site energy.

Based on the site energies (obtained here using QM/MM and the PMM methodologies, see Table 2 and Fig. 3b), the identity of the red chlorophylls in CP43 differs from the previous assignments in literature (see Table S3†). We identify the red-most chlorophyll to be in the lumenal layer of CP43, with major contributions from sites C1 (C501 in 3WU2) followed by C3 and C2. The recent computational studies by Sarngadharan *et al.*^27^ reflect the same trend of the red most chlorophylls being on C1 and C3 in the lumenal layer, consistent with the current results (Table 2). The second lowest site energies are on chlorophylls C9 and C7 from the stromal layer. This is in line with the work by Jankowiak *et al.* on non-photochemical HB studies who first reported that the two “trap” states^38^ are localized in different layers of the thylakoid membrane. Another important finding by Hughes *et al.* identified that at least one of the red chlorophylls has the 13^1^-keto group hydrogen bonded to a protein residue.^37^ Based on the 1.9 Å crystal structure, both chlorophylls C9 and C7 are H-bonded to C-Ser275 and C-His164 respectively while C1 is H-bonded to a H_2_O *via* the 13^1^-keto group. By contrast C11 (C511 in 3WU2), a proposed site for the red most chlorophyll in some assignments, is seen to possess a significantly high first excitation energy based on our calculations. This Chl has a unique axial ligation to the sidechain of PsbC-N39 and is in close contact with PsbK (see Fig. 2) and the N-terminal loop region of CP43 exposed to bulk water. Based on the PMM results in Table 2, the site energy of C11 has a significantly high standard deviation, which denotes that changes in the local protein environment induce large fluctuations in the computed site energies. There have also been discrepancies regarding the site energy of chlorophyll C4 (C504 in 3WU2). Some studies based on refinement fits of optical spectra identified C4 to have a low excitation energy,^28^ but our findings as well as previous structure-based simulations on the CP43 subunit do not agree with this assignment. However, it is also important to note that the inclusion of inter-pigment interactions, particularly electronic and excitonic couplings between specific chlorophylls or groups of chlorophylls may change the relative trends in site energies.

At this point it is important to consider the above results in relation to the D1 protein. The peripheral chlorophylls Chl*z*_D1_ and Chl*z*_D2_ are discussed in the context of EET from the CP43 and CP47 core antennae, respectively, to the RC. Even though these two pigments are bound to the D1 and D2 proteins, they are thought to be functionally associated to the CP43/CP47 core antennae.^18^ Notably, Chl*z*_D1_ is in van-der-Waals contact (4.2 Å distance) with a β-carotenoid (Car_D1_) to which it can be excitonically coupled, and is close to the D1 active branch RC pigments (Fig. 2, *ca.* 21 Å), and hence assumed to play a direct role in EET between CP43 and the RC. The recent study by Yang *et al.* reported a faster EET from CP43 to Chl*z*_D1_ compared to EET from Chl*z*_D1_ to other D1 pigments,^19^ concluding that the latter process is the actual rate limiting step in the overall EET pathway, consistent with previous studies by Renger and co-workers.^42^ Nguyen *et al.* performed similar studies on D1D2Cyt_b559_ complexes and identified a distinct excitonic state prior to primary charge separation (Trap*) likely belonging to one of the Chl*z* pigments.^97^ The above findings are not in agreement with the theoretical study of Hsieh *et al.* who reported that the Chl*z* sites do not mediate EET into the RC based on molecular dynamics simulations of the PSII core complex.^98^ Here, we explicitly compare the excited state energetics of the peripheral D1 chlorophyll Chl*z*_D1_ with the CP43 chlorophylls, including the full effect of PSII protein electrostatics (QM/MM) and conformational changes (PMM). Based on our results, Chl*z*_D1_ exhibits a lower Q_*y*_ (S_0_–S_1_) excitation energy (1.839 eV) than all CP43 chlorophylls. Even with the inclusion of the dynamics based on PMM calculations, we find that Chlz_D1_ has a consistently lower mean site energy (1.855 eV) than all other CP43 chlorophylls. Based on our findings, we cannot explicitly conclude if Chl*z*_D1_ can mediate EET from CP43 to the RC. Also, we do not locate the lowest site energy on the nearest CP43 pigment, C5. Nevertheless, one possible implication could be that the low-lying excited state on the peripheral Chl*z*_D1_ may act as a protective “trap” state to quench excess excitation energy from the RC, and avoid photodamage of the D1 protein.

### 3.2. Excitonic couplings from PMM

In light harvesting complexes (LHC), the site energies of individual chromophores can be effectively calculated by means of TD-DFT methods as shown in the previous section. However, in most cases treating groups of chromophores at one QM level may be challenging. Consequently, most computational studies of LHCs employ a combination of high-level methods to compute site energies of the individual Chls and use more simplified theoretical models to approximate the coupling interactions between them.^51^ The coupling terms gives an estimate of the interaction between a pair of electronic excitations localized on different Chls and together with site energies is necessary to build a complete excitonic model of light harvesting proteins.^18,99,100^ In this work, the excitonic couplings between Q_*y*_ transitions of Chls in CP43 were calculated using the PMM approach on the QM/MM optimized geometries and a modified version of the point dipole approximation (PDA) method described earlier (see Section 2.3).

The calculated excitonic coupling values are listed in Table 3. The largest couplings mainly involve the following pigment pairs: C2–C4, C8–C9, C9–C11, C7–C9, C5–C7, C10–C11, C6–C7 and C2–C10. Except for the C5–C9 dimer, all other couples exhibit relatively small center-to-center distances (see Table S5†). The chlorophylls C7, C9 and C10 are the most strongly coupled, *i.e.*, they strongly interact with at least three other chlorophyll pairs with significantly high coupling values. The largest excitonic couplings (*i.e.*, with absolute values greater than 100 cm^−1^) are found for the C2–C4, C8–C9, C9–C11 and C7–C9 pairs.

**Table 3:** Excitonic coupling constants between CP43 chlorophylls computed using the MD-PMM approach. All values are reported in cm^−1^. Absolute values greater than 50 cm^−1^ are shown in bold. The corresponding standard deviations are shown in Table S4

	C2	C3	C4	C5	C6	C7	C8	C9	C10	C11	C12	C13
C1	10.89	21.90	3.09	−3.07	−12.22	21.05	7.15	10.89	2.70	−2.52	0.63	−0.11
C2		8.14	**154.07**	−8.67	−16.15	−1.01	16.94	23.26	**73.09**	5.05	12.33	−10.27
C3			−36.43	4.48	−0.45	7.88	−4.39	−11.26	23.04	8.19	25.02	5.71
C4				−3.40	4.57	−2.08	−1.53	−0.97	−34.68	−4.46	−4.72	5.09
C5					25.46	**−98.32**	−5.90	−31.38	−1.18	−0.81	−1.55	5.03
C6						**−77.69**	−1.14	−32.26	−12.14	−4.85	−4.15	10.23
C7							17.84	**−112.59**	−4.61	−9.06	−34.46	26.34
C8								**130.37**	**66.94**	1.78	2.11	−5.16
C9									16.20	**−117.64**	36.43	−6.15
C10										**−97.23**	19.40	−11.76
C11											25.24	−13.91
C12												−48.50

The only exception in our estimated excitonic coupling values compared to those in previous literature is seen for the C2–C10 chlorophyll pair which yields a high excitonic coupling value of 73 cm^−1^. Moreover, the location of the C2–C10 is such that it connects the two layers of pigments in CP43 (Fig. 2c). A high excitonic coupling constant therefore implies that this dimer may play a role in EET between the stromal and lumenal layers of CP43. Similar arguments were made by Saito *et al.* in recent computational studies of EET coupling in the PSII-cc.^61^ It is to be noted that excitonic coupling values in closely spaced pigments are highly sensitive to the approximations used to estimate them.^101^ For instance, here the point dipole approximation is seen to predict a large positive coupling for the C2–C10 pair, but the TrEsp method in past investigations reported small coupling values for the same pair.^27,29^

It is noted that the couplings reported here are slightly higher in magnitude compared to those reported in previous theoretical studies,^27,29^ but are of the same magnitude as the values estimated based on PDA by Ishikita and co-workers on CP43 (ref. 61) and those by Grondelle and co-workers on the PSI-Lhca4 complex.^95^ This trend may be attributed to several effects, namely the overestimation of gas-phase transition dipoles by TD-DFT,^41^ or the fact that in the current implementation of the PMM procedure the perturbation does not include polarization effects of the environment,^50,96,97^ which may be responsible for a screening of the couplings. Consequently, in order to account for delocalized excited states within strongly coupled groups of pigments, one likely needs to employ more accurate quantum chemical methodologies.

### 3.3. Excited states of chlorophyll dimers

In photosynthetic light harvesting complexes and RC proteins, closely spaced pigments possess excited states that are electronically or excitonically coupled to each other. Although individual site energies and excitonic couplings already reveal a lot about the excitonic manifold of light harvesting pigments, understanding the mechanism of EET within the core antennae as well to and from the RC additionally requires direct insight into the excitation profiles of multiple coupled chromophores. In the case of CP43, Müh *et al.* reported two degenerate low-energy exciton transitions that represent the lowest excited states of the two “domains” in the lumenal (containing C2 and C4) and stromal layers (containing C5 and C7–C11).^28^ Earlier studies based on Stark and triplet-minus-singlet (T − S) spectra^31^ provided evidence about partially delocalized excited states in the CP43 antenna as well, but no investigations exist so far to directly describe short-range effects on the excited states of pigment pairs in the CP43 or CP47 core antennae. Toward this objective, we computed the low energy excited states for specific chlorophyll pairs within the “intact” CP43 in PSII-cc. The pigment pairs in CP43 were selected based on the following criteria: (a) center-to-center (Mg–Mg) distances less than 10 Å (b) the excitonic couplings calculated here (see Table 3). The nature of the excited states for each Chl dimer (C2–C4, C2–C10, C5–C7, C6–C7, C7–C9, C8–C9, C8–C10, C9–C10, C9–C11, C10–C11 and C12–C13) is characterized based on analysis of the natural transition orbital (NTO) coefficients for each transition. The TD-DFT results are summarized in Table S6 of the ESI.†

The four Chl pigments C1–C2–C3–C4 constitute the lumenal layer of CP43 with an average Mg–Mg distance of 12.3 Å (see Fig. 2c). As discussed in Section 3.1 based on our TD-DFT and PMM results on individual pigments, we identified C1 and C3 to possess the lowest site energies in the “intact” CP43 antenna. C2 and C4 are the most closely arranged pigments in this layer and our findings suggest that this pigment pair has a strong positive coupling constant (154 cm^−1^), consistent with previous studies.^27,29,61^ However, based on our calculations, their individual site energies are not isoenergetic and our calculations (Table S6†) on the C2–C4 dimer do not reveal any delocalized exciton. The energy corresponding to the lowest excited state on C2 remains unaffected, but the exciton localized on C4 is only slightly red-shifted (29 meV).

**Fig. 4:**
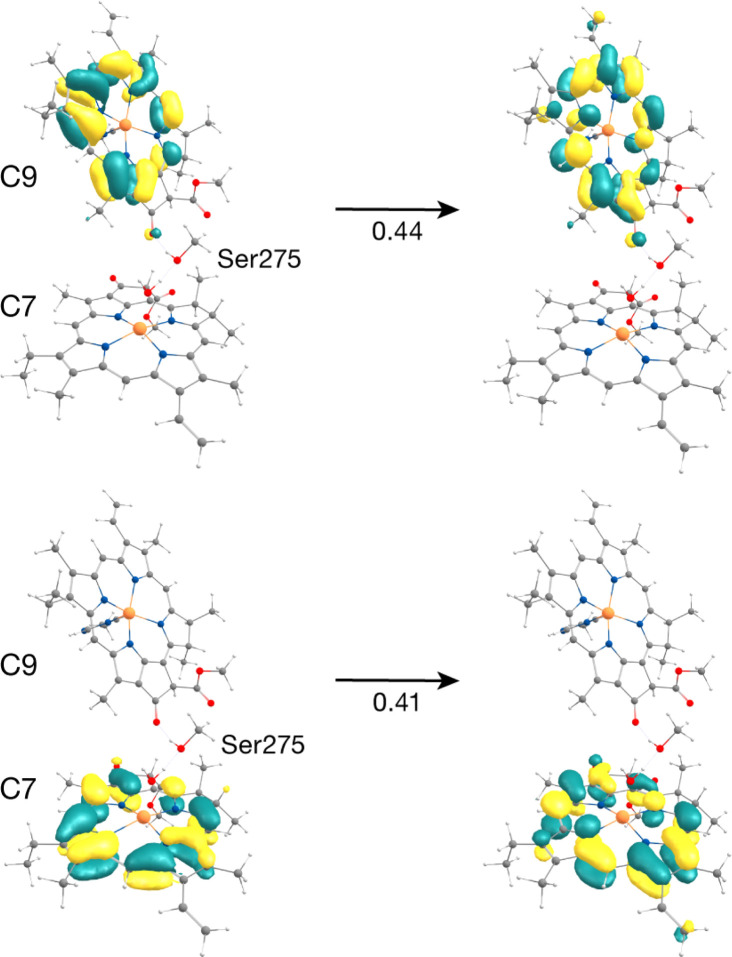
Donor–acceptor NTOs for the S_1_ state (1.868 eV) of the C7−C9 dimer in the “crystal-like” snapshot, demonstrating that the state has mixed local excitation character, with approximately the same coefficients for the two excitations.

The C7–C9 pair is in the stromal layer of CP43 with an Mg–Mg distance of 11.4 Å. Interestingly, both lowest excited states S_1_ (1.868 eV) and S_2_ (1.891 eV) are superpositions of the Q_*y*_ transitions of C9 and C7 (analogous to the P_D1_P_D2_ special pair in the RC).^62^ The NTO pairs for the corresponding S_1_ and S_2_ transitions are shown in Fig. 4. The delocalized lowest excited state (S_1_) for this dimer is seen to possess an even lower excitation energy while the S_2_ state has a slightly higher energy compared to the similar Q_*y*_ energies of the individual pigments C9 and C7 (1.881 and 1.885 eV respectively). The further red-shift of the lowest excited state in the dimer allows us to conclude that C9 and/or C7 may contribute majorly and almost equally to the red trap state in the stromal layer of CP43. This is in line with previous assignments of the lowest excitonic states made by Renger and coworkers.^28,29^ Based on the S_0_ → S_1_ excitonic couplings reported by Müh *et al.*,^28^ Saito *et al.*^61^ and Sarngadharan *et al.*,^27^ and those obtained in this work using the MD-PMM approach, both C7 and C9 are part of a strongly coupled group of chlorophylls in the stromal layer. The low energy excitation profiles of the remaining Chl pairs in the cytoplasmic layer (C5–C7, C6–C7, C8–C10, C9–C10, C9–C11, C10–C11, C12–C13) majorly constitute localized excitations corresponding to the Q_*y*_ and Q_*x*_ transitions on individual pigments.

One motivation for the study of pigment dimers is the identification of possible charge transfer states.^102–109^ In photosynthetic pigment–protein complexes CT states may participate in either spectral-tuning^103^ or photoprotection through the rapid quenching of excess excitation energy.^104^ It has been suggested that the latter process likely involves an excitation energy transfer from an excited Chl monomer to a strongly coupled Chl leading to a rapid non-radiative decay process *via* short-lived intermediate CT states.^105^ Fleming and co-workers reported CT quenching in the minor antenna complexes of PSII (CP29, CP26 and CP24).^106,107^ Ramanan *et al.* have reported the presence of mixed excitonic-CT states in Chl heterodimers in the low-energy manifold of the LHCII complex.^108^ More recently, Ostroumov *et al.* reported the presence of far-red emitting Chl–Chl CT states as intermediates in the excited state quenching of LHCII.^109^ Stark spectroscopy and more recent computational studies on another light harvesting protein, the PSI-Lhca4 complex demonstrated that a distinctive red-shifted emission originates from the mixing of the lowest exciton state with a CT state of an excitonically coupled dimer.^110^ Intermolecular charge-transfer (CT) states among photosynthetic pigments (Chl_D1_^+^Pheo_D1_^−^ or P_D1_^+^Pheo_D1_^−^) directly control primary charge separation and charge recombination processes in the PSII-RC,^26,63,65^ but the presence of CT states in CP43 or CP47 core antenna proteins of PSII has not been reported. The present TD-DFT calculations on Chl dimers enable us to approach this question for the case of CP43.

**Fig. 5:**
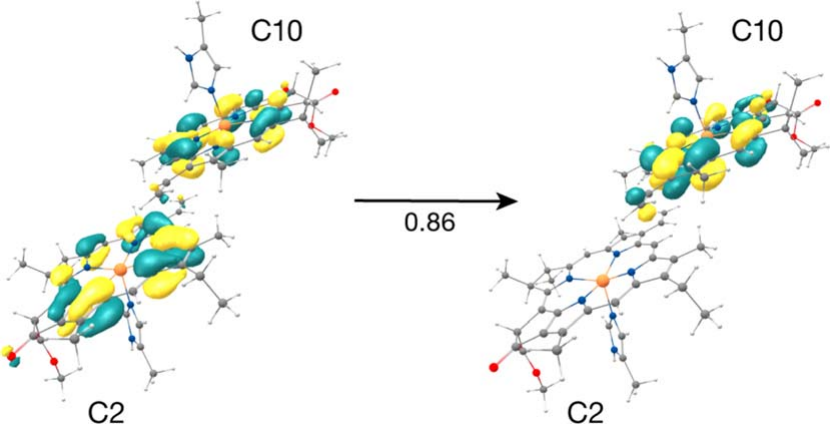
Donor–acceptor NTOs for the S_2_ state (1.943 eV) of the C2–C10 dimer in the ‘crystal-like’ snapshot, which shows considerable C2 → C10 charge-transfer character mixed with the local excitation on C10.

The result that stands out concerns the C2–C10 pair. These chlorophylls (Fig. 5, Mg–Mg distance 10.2 Å) constitute a stacked dimer with parallel ring planes located approximately in the center of the CP43 transmembrane region connecting the stromal and lumenal layers (see Fig. 2c). Our results on the “crystal-like” configuration (snapshot 1) indicate that the first excited state for this dimer is localized on C2 (S_1_, 1.843 eV), a likely candidate for the lowest excitation energy in CP43. Most importantly, we find the second S_2_ (1.943 eV), and third (S_3_, 2.001 eV) excited states of the C2–C10 dimer to have C2 → C10 CT character mixed with the lowest excited state of C10. A detailed analysis of the NTOs involved in the transition *i.e.*, the precise decomposition of the transition based on the NTO coefficients, shows that the second (S_2_) and third (S_3_) excitations are represented by a “delocalized” donor orbital within the C2–C10 dimer, with the acceptor NTO localized on C10, resulting in substantial CT character. The NTOs for the S_2_ state are shown in Fig. 5.

Until now we looked into the excited state properties of the Chl dimers of CP43 based on a single “crystal-like” structural configuration of PSII (snapshot 1). Now we investigate how the dynamics of PSII influence the CT excitation character in the C2–C10 pair. For this purpose, we performed the same type of excited state calculations, each with individually optimized QM/MM geometries, on nine additional structurally independent snapshots (snapshots 2–10) obtained from unbiased production MD with consecutive intervals of 5 ns. The QM/MM-TDDFT results on the C2–C10 pair are provided in Table S7.† Fig. S5 and S6† depict the NTOs and difference densities respectively for the lowest CT state of the pair, which shows that the effect of the protein matrix is similar both in the crystal-like conformation of the protein and in the selected MD snapshots. The relative order of site energies remains the same, *i.e.*, Chl C2 has a lower site energy than C10, but the C2 → C10 CT character is distributed differently among the S_1_, S_2_ and S_3_ states depending on the protein configuration. Specifically, in the crystal-like snapshot both S_2_ and S_3_ states have CT character mixed with local excitation on C10 while in half of the selected MD snapshots the S_3_ state has dominant C2 → C10 CT character (see Table S7†). Interestingly we also find that the S_1_ state has some CT character mixed with the local excitation on C2 for two of the examined protein configurations. Our findings thereby demonstrate that the conformational dynamics of PSII tune the extent of LE–CT mixing of the C2–C10 pair in its lowest excited states and thus allows the C2 → C10 CT states to span an energy range that can bring considerable CT character as low as 1.81 eV. Such dynamic evolution of CT character has also been demonstrated in the low-energy excitation profile of RC pigments.^26,62,64,65^ The C2–C10 dimer is not the only Chl pair in CP43 to possess a CT state; our results locate higher-energy excited states (above 3 eV) with pure CT character for the C9–C11, C9–C10, C8–C10, C10–C11 pairs (Table S6†), however the C2–C10 pair is unique in having a very low-lying CT state, essentially interleaved with the lowest locally excited states of the whole system.

Understanding the molecular mechanism of formation of low-lying CT states in LHCs has crucial implications not only for mechanisms of light harvesting and EET but also for photoprotection. Recently, Sláma *et al.* employed multiscale modelling approaches to show that the low-lying red states and red-shifted fluorescence bands in PSI-Lhca4 originate from the interplay of exciton and CT states within a Chl pair.^110^ Such energetically low-lying mixed excitonic-CT states have also been assigned to Chl heterodimers in the excitonic manifold of the major plant light harvesting complex LHCII based on 2DES studies.^108^ Mixed exciton-CT states may also act as an energy sink and thus determine pathways of EET in normal light conditions, or in some cases, may participate as intermediate trap states for excitation energy quenching in excess light.^109^ Raszewski and Renger^41^ in their seminal work on the PSII core complex antennae proposed that CP43/CP47 may switch from a “light-harvesting” mode for open RCs to a “photoprotective” mode for closed RCs. Therefore, our identification of a C2 → C10 CT state coupled to the excitonic manifold of C10 may have the following functional implications: (a) facilitate EET within CP43 from the lumenal to the stromal layer, (b) spectral tuning of the “low-lying” red chlorophylls in CP43, or (c) act as intermediate “trap” states for quenching of excess excitation energy away from the RC. The latter is particularly relevant for CP43 owing to its close proximity to the RC pigments and the D1 protein.

### 3.4. Global CP43 excitonic states from the MD-PMM approach

**Fig. 6:**
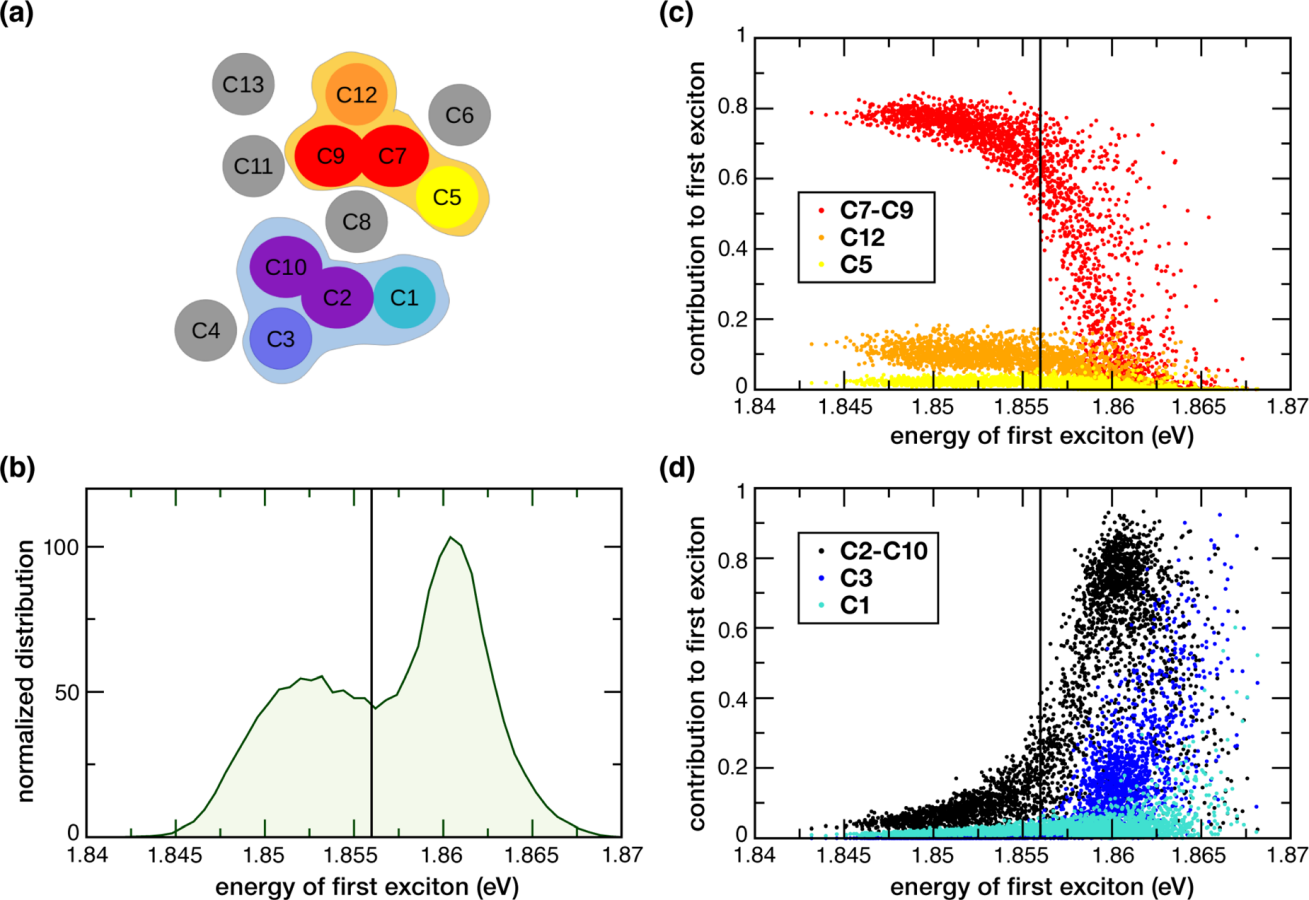
(a) Chlorophyll network in CP43. Two groups of pigments are identified according to their contribution to the first excitonic state. The pigments highlighted in light orange contribute to the lowest-energy subpopulation, while the ones in light blue to the second lowest-energy subpopulation. (b) Energy distribution along the MD simulation of the first excitation energy of the first excitonic state (first exciton). (c) Contribution of C5 (yellow), C12 (orange) and the C7−C9 dimer (red) to the first exciton as a function of the first excitation energy for each MD configuration. (d) Contribution of C1 (cyan), C3 (blue) and the C2−C10 dimer (black) to the first exciton as a function of the excitation energy for each MD configuration.

In the previous section we examined excited states of selected Chl dimers using a static TD-DFT approach. Here we report the global excitonic states considering all 13 Chls in CP43 by means of the MD-PMM procedure using as basis the single chlorophylls for C1, C3, C4, C5, C6, C8, C11, C12 and C13 and the two dimers, C7–C9 and C2−10. The C7–C9 and C2–C10 dimers, instead of the single C2, C7, C9 and C10 chlorophylls, were used because these pairs of pigments showed a non-negligible electronic coupling and distinctive nature of the excited states (see Sections 3.2 and 3.3). A schematic representation of the spatial arrangement of the pigments is shown in Fig. 6a. The distribution along the MD simulation of the vertical transition energies of the first excitonic state is shown in Fig. 6b. It is important to note that the low-energy exciton is not always localized on the same chlorophyll sites. We found two red-shifted trap-states that change depending on conformational fluctuations: one at lower energy (1.852 eV on average) with contribution from C7–C9 dimer, C12, C5, the other at slightly higher energy (1.860 eV on average) with contribution from C2–10 dimer, C3 and C1.

The lowest exciton shows two subpopulations that depend on fluctuations in the protein conformation, which implies two major conformational basins with slightly different excitonic energies. The analysis of the contribution of the pigments and dimers used as basis for the excitonic coupling calculations shows that the C7–C9 and C2–10 dimers contribute the most. Minor contributions arise from Chls C1, C3, C5, C8 and C12. Most importantly, two rather distinct groups of coupled pigments participating to the two subpopulations mentioned above, can be identified: the one at lower excitonic energy (peaked at around 1.852 eV) characterized by the contributions from the C7–C9 dimer, C12 and, as minor contribution, C5 and the other (peaked at around 1.860 eV) characterized by contributions from the C2–C10 dimer, C3 and, as minor contribution, C1 (the C8 contribution is not mentioned because it provides a similar contribution to the two groups). The weight of the contributions of the different monomeric and dimeric pigments to the first exciton is highlighted in Fig. 6. The C7–C9 dimer provides the major contribution (with a weight of around 0.8) to the most red-shifted trap, along with C12 (with a weight of around 0.12–0.15) and C5 (with a weight of around 0.08–0.05) (see Fig. 6c). Instead, The C2–C10 dimer provides the major contribution (with a weight in the range of 0.6–0.8) to the second trap, along with C3 (with a weight in the range of 0.10–0.30) and C1 (with a weight of around 0.05–0.10) (see Fig. 6d). In one investigation, Müh *et al.*^28^ reported that two degenerate low-energy exciton transitions represent the lowest excited states of the two domains in the lumenal (containing C2 and C4) and stromal layers (containing C5 and C7–C11). Our results here support a similar scenario, in the sense of two excitonic domains in the lumenal and stromal layer, but with different contributions from the different pigments. It would nevertheless be interesting to have an estimate of the EET kinetics from the second trap (C2–C10, C3, C1) to the first (mostly red-shifted) trap (C7–C9, C12, C5), but this remains beyond the scope of the current work.

Based on our results, 3 out of the 4 Chls with the lowest site energies *i.e.*, C3, C7, C9 (Fig. 3b) are located in the center of each layer. Interestingly, these pigments show strong excitonic coupling with each other group of chlorophylls (Table 3). For instance, C9 is strongly coupled to C7 (−112.59 cm^−1^) which is excitonically coupled to C5 (−98.32 cm^−1^). We found that this pair possesses delocalized excited states (Fig. 4) in the low-energy regime, and contributes almost equally and majorly to the first excitonic state of CP43 (Fig. 6c). This identity of the low-energy trap state agrees with earlier assignments made by Müh *et al.*^28^ and Shibata *et al.*^16^ suggesting that chlorophylls C9 and C7 belong to the same excitonic domain, as also seen in this work. Although some studies claim that the low energy sinks in the CP43/CP47 core antenna should be in close proximity to the RC pigments to facilitate efficient EET, this argument remained controversial because some simulations suggested that EET from the stromal layer to the RC is equally efficient as EET from the lumenal layer.^41^ Towards this, our assignment of a coherent excitonic domain comprising C7, C9 and C5 in the stromal layer has important functional implications towards EET from CP43 to the RC because C5 is indeed the closest CP43 pigment to peripheral Chl*z*_D1_ as well as the RC pigments (Chl_D1_ and Pheo_D1_). This finding can be considered to be in agreement with recent studies by Yang *et al.*^19^ because we identify C5 as part of the lowest excitonic domain in C43 and therefore as a pigment that can mediate energy transfer from CP43 to the RC.

Our calculation of the global excitonic states reveals another distinct excitonic domain comprising chlorophylls C1, C3 and the C2–C10 dimer (Fig. 6d). Our TD-DFT results on C2–C10 indicated a low-lying CT state mixed with the excited state of C10 (Fig. 5). While recent computational studies have investigated the role of mixed excitonic-CT states towards the spectral tuning of “red” fluorescence states in PSI-Lhca4,^110^ the involvement of a CT state in spectral tuning of the “red” states in PSII core antenna complexes have never been reported. Based on our current results, the C2–C10 interaction red-shifts the Q_*y*_ energy of C2 by *ca.* 24 meV, and most importantly this pair also forms a coherent excitonic domain along with the other “red” pigments C3 and C1 (Fig. 3b). Both results imply that the C2 → C10 CT state likely plays a role in the spectral tuning of the “red” trap state within the lumenal layer of CP43.

## 4. Conclusion

In this work, we investigated the low-energy excitation manifold of the CP43 core antenna in PSII, utilizing a multiscale TD-DFT QM/MM approach combined with large-scale MD-PMM calculations. The site energies computed with both TD-DFT and PMM provide a qualitative agreement for the red-most pigments being distributed in two groups: C7 and C9 in the stromal, C1 and C3 in the lumenal layer. The excitonic couplings derived using PMM facilitated the identification of specific Chl pairs that exhibit strong inter-pigment interactions. TD-DFT calculations on selected chlorophyll pairs revealed a delocalized excited state on the C7–C9 dimer. Notably, calculations on the C2–C10 dimer identified a low-lying charge-transfer state mixed with the local excitation on C10. Further studies will be required to understand the physiological significance of this finding.

Finally, we reported global excitonic states involving all 13 Chls in CP43 by means of the MD-PMM procedure. Our findings led us to conclude that the lowest excited state is not localized on the same chlorophyll site at each system configuration but its nature depends on the dynamics of the protein matrix. Specifically, we find two red-shifted excitonic domains, one in each layer of the thylakoid membrane. The lower energy excitonic state has contributions from C7–C9, C12 and C5. Although unique, this identity of the low-energy trap state aligns in several ways with past assignments.^16,19,28^ The concept of domains has been previously discussed in the literature, but our results present a new perspective. We demonstrate that the identity of these domains is influenced by conformational motions, and their relative energy can be thus modulated through conformational dynamics. This is particularly significant at room temperature, where conformational dynamics play a crucial role.

The coherent excitonic domain in the stromal layer (comprising C7, C9 and C5) has functional implications for EET from CP43 to the RC because C5 is the closest CP43 pigment to peripheral Chl*z*_D1_ as well as the RC pigments.^19^ The other excitonic domain involves the C2–C10 dimer along with C3 and C1 in the lumenal layer. Most importantly, the mixed excitonic-CT state on C2–C10 may play a role in the spectral tuning of the “red” pigments in the lumenal layer of CP43 or act as intermediate for quenching of excess excitation energy in PSII (photoprotection). Overall, this study establishes a refined basis for future kinetic modelling of EET pathways as well as for structure-based interpretation of spectroscopic properties of CP43, and contributes to an improved understanding of light harvesting and excitation energy transfer in oxygenic photosynthesis.

## Data availability

Original data from this work are provided as an open-access data set hosted by the Open Research Data Repository of the Max Planck Society at https://doi.org/10.17617/3.S2DPUE.

## Author contributions

S. B.: investigation, analysis, writing – original draft; S. A.: investigation, analysis; I. D.: methodology, supervision, writing – original draft, writing – review and editing; D. A. P.: conceptualization, methodology, supervision, writing – review and editing.

## Conflicts of interest

There are no conflicts to declare.

## Supplementary Material

SC-015-D3SC06714A-s001

SC-015-D3SC06714A-s002
