# Supplementary material for: Excitation landscape of the CP43 photosynthetic antenna complex from multiscale simulations
Source: Chem Sci. 2024 Apr 9;15(19):7269–84. doi: 10.1039/d3sc06714a (PMC11095388; doi:10.1039/d3sc06714a)
Supplement: SC-015-D3SC06714A-s001 [file SC-015-D3SC06714A-s001.pdf]

Electronic Supplementary Material (ESI) for Chemical Science.
This journal is © The Royal Society of Chemistry 2024

Excitation landscape of the CP43 photosynthetic antenna complex from multiscale simulations

Sinjini Bhattacharjee[a], Srilatha Arra[b], Isabella Daidone[*b], Dimitrios A. Pantazis[*a]

[a] Max-Planck-Institut für Kohlenforschung,
Kaiser-Wilhelm-Platz 1, 45470 Mülheim an der Ruhr, Germany

[b] Department of Physical and Chemical Sciences, University of L'Aquila,
via Vetoio (Coppito 1), 67010 L'Aquila, Italy

ELECTRONIC SUPPLEMENTARY INFORMATION

Additional Computational Details

Molecular Dynamics (MD) simulations

The initial structure of the Photosystem II (PSII) monomer used in this work is based on the high-resolution crystal structure of *T. vulcanus* (PDB ID: 3WU2).[1] The complete protein unit was embedded in a POPC lipid bilayer of dimension 176 × 176 Å^2 using Packmol-Memgen,[2] thoroughly solvated with a TIP3P water box (**Figure S1**) and neutralized with appropriate number of counterions in a 0.15M NaCl salt buffer. For the protein residues, waters and POPC bilayer we used the standard parameters from the AMBERff14SB,[3] TIP3P[4] and LIPID17[5] force fields, respectively. The partial charges and force field parameters for the organic cofactors (plastoquinones, carotenoids, structural lipids) were obtained using GAFF2,[6] bonded parameters for non-heme Fe and OEC were taken from earlier studies,[7-9] while those of the remaining cofactors (Chl a, Pheo, heme) were obtained from literature.[10, 11] The nonbonded parameters for the metal ions were based on their respective oxidation states using data sets available for the TIP3P water model.[12] For Na^+ and $Cl^–$ ions, we used the Joung–Cheatham parameters compatible with the TIP3P water model.[13, 14]

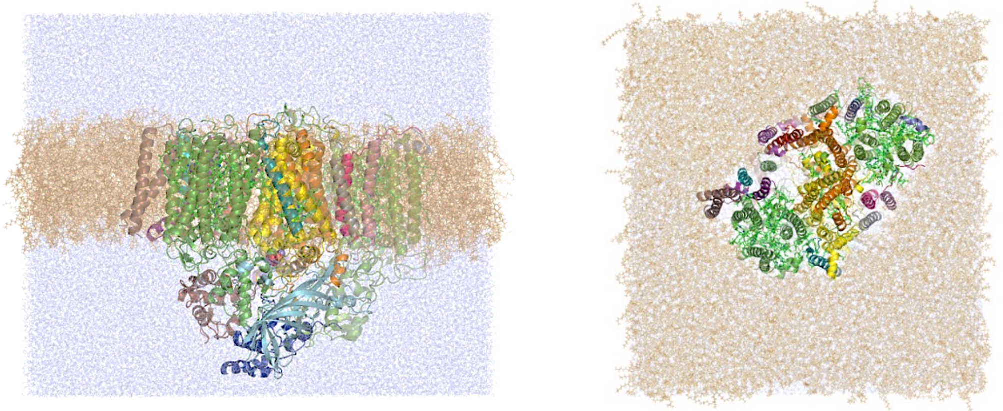

Figure S1. Side view and stromal (top) view of the molecular-mechanics (MM) model of the lipid bilayer bound PSII monomer used in this work (Na^+ and $Cl^–$ counterions not shown for clarity).

A step-by-step minimization protocol was employed to remove energetically unfavorable geometric clashes in the system. In the heating phase, the system is first heated from 10 to 100 K in a succession of 5 ps in the NvT ensemble, followed by further heating from 100 to 303 K in the NpT ensemble for a total of 125 ps. The temperature during this step is maintained using the Langevin dynamics[15] with a collision frequency of 5 ps$^{–1}$. During the equilibration phase, the C_α atoms of amino acids were restrained with a force constant of 20 kcal mol$^{–1}$Å$^{–2}$. Subsequently, the restraints on the C_α atoms were systematically reduced (2 kcal mol$^{–1}$Å$^{–2}$/500

ps) and the system was further equilibrated for 65 ns to equilibrate the POPC bilayer. Thereafter, production run was initiated without any restraints and the temperature and pressure set at 303 K and 1 atm, respectively. During the entire procedure, the temperature was controlled using Langevin dynamics with a collision frequency of 1 $ps^{-1}$and the system pressure was controlled using the Berendsen barostat[16] with anisotropic pressure scaling with a relaxation time of 2 ps. We employed the SHAKE algorithm[17] to constrain the bonds involving hydrogens, therefore a time step of 2 fs could be used. The electrostatic interactions were treated using the Particle Mesh Ewald (PME) approach[18] with a 10 Å cut-off. The AMBER20 package[19] was used to perform the energy minimizations and equilibration dynamics while the production MD simulations were performed in the GPU version of the pmemd module (*pmemd.cuda*).[20]

Calculation of the absorption spectrum with the MD-PMM approach

For computing the absorption spectrum, after calculating the perturbed frequencies (ν) and transition dipoles ($\boldsymbol{\mu}_{j,i}$) for each exciton state over the frames of the MD trajectory, we evaluate the excitation energy distribution using a suitable number of frequency bins. Subsequently, we utilize the excitation energy distribution and the corresponding transition dipoles to determine the molar extinction coefficient $\varepsilon_{0,i}$ for the transitions from the ground to the ith excited state, thus providing the absorption spectrum according to:

$$\epsilon_{0,i} = \sum_{\nu_{\mathrm{ref}}} \frac{\Gamma_A(\nu_{\mathrm{ref}}) h\nu}{N} \frac{e^{-(\nu-\nu_{\mathrm{ref}})^2/2\sigma^2}}{\sigma\sqrt{2\pi}} \quad \text{(S1)}$$

$$\Gamma_A(\nu_{\mathrm{ref}}) = \frac{\left|\boldsymbol{\mu}_{0,j}\right|^2_{\nu_{ref}}}{6\epsilon_0 c\hbar^2} \quad \text{(S2)}$$

Here, ν_{ref}, $n(\nu_{\mathrm{ref}})$ and $\left|\boldsymbol{\mu}_{0,j}\right|^2_{\nu_{ref}}$ are the frequency at the center of each bin, the corresponding number of MD frames and mean transition dipole square norm within the bin, respectively, $\hbar = h/2\pi$ with h the Planck constant, ϵ_0 is the vacuum dielectric constant, c is the speed of light and σ^2 is the variance associated to the semiclassical intra-QC vibrations neglected in the evaluation of the unperturbed properties. In the present case, the value of σ^2 ($\sigma = 10^{-4}$ Hartree) has been estimated on the basis of the full width at half maximum (FWHM) of the experimental spectrum of chlorophyll a in toluene,[21] approximating the vacuum condition. The calculated spectral line shape in the Q_y region is reported in **Figure S2**. Note that the width of the

calculated bands (inhomogeneous broadening) is due to the explicit effect of the semiclassical fluctuations of the QCs and their environment along the MD trajectory and does not include any adjustable parameter.

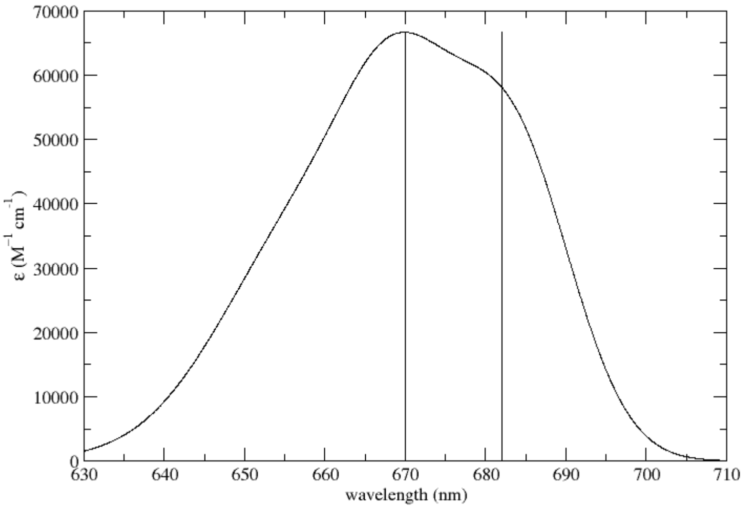

Figure S2. Room-temperature absorption spectrum of the CP43 complex calculated with the MD-PMM approach. The spectrum has been shifted by 0.000457 Hartree to lower energies in order to match the position of the main experimental peak measured at 293 K.

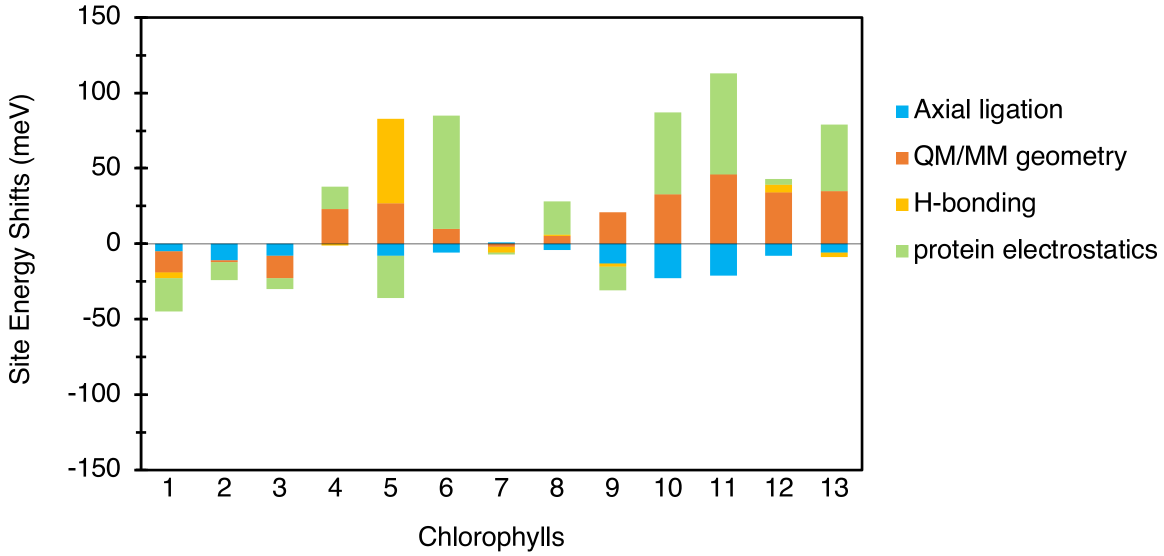

Figure S3. Decomposition of the electrostatic and non-electrostatic contributions (axial ligation, H-bonding, QM/MM geometry optimization) to the site energy shifts of the CP43 chlorophylls.

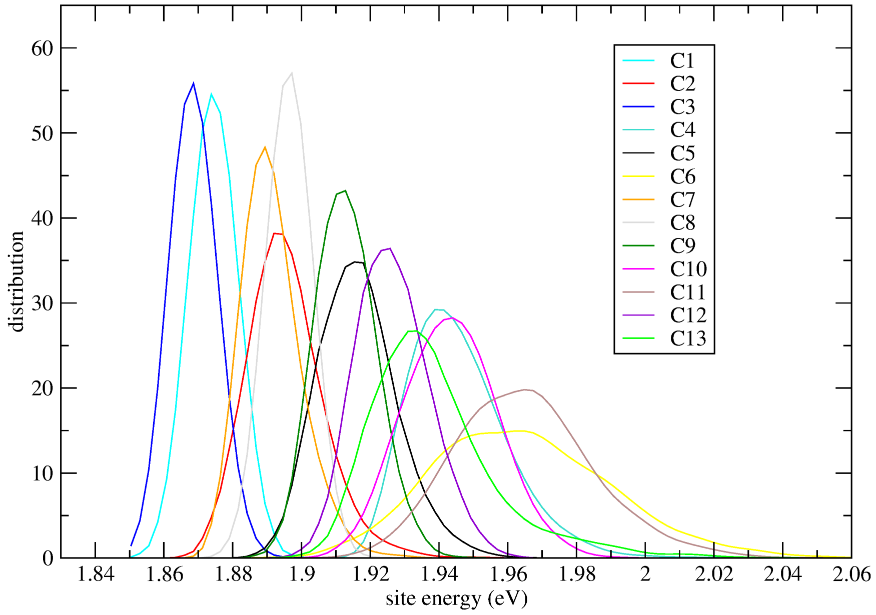

Figure S4. Distributions of the site energy for all Chl-a molecules (which include their respective axial ligands) in the CP43 complex performing the MD-PMM calculations over the last 30 ns of the MD trajectory (3000 frames in total).

Table S1. TD-DFT (ωB97X-D3(BJ)/def2-TZVP) excitation energies *E* (in eV) of the S_2/Q_x states and corresponding oscillator strengths for the CP43 chlorophylls computed *in vacuo*, with QM/MM on an isolated CP43 protein, and with QM/MM in the complete PSII monomer. The geometries in all cases are derived from QM/MM optimizations within the PSII monomer. Shifts are reported in meV with respect to the gas-phase values.

	**TD-DFT Gas-phase**		**TD-DFT | QM/MM Isolated CP43**			**TD-DFT | QM/MM Full PSII monomer**		
**Site**	***E***	f_{osc}	***E***	f_{osc}	**Shift**	***E***	f_{osc}	**Shift**
**C1**	2.354	0.04	2.296	0.03	-58	2.260	0.02	-94
**C2**	2.372	0.04	2.307	0.03	-65	2.280	0.02	-92
**C3**	2.370	0.02	2.303	0.02	-67	2.283	0.01	-87
**C4**	2.427	0.03	2.359	0.01	-68	2.375	0.02	-52
**C5**	2.475	0.16	2.417	0.12	-58	2.440	0.15	-35
**C6**	2.383	0.06	2.457	0.09	74	2.464	0.11	81
**C7**	2.421	0.05	2.392	0.04	-29	2.382	0.05	-39
**C8**	2.394	0.05	2.412	0.03	18	2.452	0.07	58
**C9**	2.404	0.06	2.354	0.06	-50	2.342	0.06	-62
**C10**	2.362	0.07	2.424	0.11	62	2.450	0.12	88
**C11**	2.371	0.07	2.473	0.18	102	2.468	0.17	97
**C12**	2.418	0.08	2.433	0.09	15	2.409	0.09	-9
**C13**	2.423	0.07	2.413	0.08	-10	2.449	0.12	26

Table S2. TD-DFT Transition Dipole moments of the CP43 chlorophylls *in vacuo*.

	S_0–S_1					S_0–S_2				
**Chl**	**T (debye)**	**T^2 (au^2)**	**T_x (au)**	**T_y (au)**	**T_z (au)**	**T (debye)**	**T^2 (au^2)**	**T_x (au)**	**T_y (au)**	**T_z (au)**
**C1**	5.4223	4.5507	1.6598	0.9657	0.9292	1.7877	0.4947	-0.0116	-0.3280	-0.6220
**C2**	5.5418	4.7535	0.7559	1.9560	-0.5969	1.9422	0.5839	0.0872	-0.4145	0.6360
**C3**	5.6286	4.9036	1.6110	-1.4899	-0.2976	1.6609	0.4270	-0.2455	0.2258	0.5619
**C4**	5.5886	4.8341	-1.4121	-1.5491	-0.6636	1.8808	0.5475	0.1257	0.3485	0.6405
**C5**	5.5772	4.8146	1.3595	-1.2380	-1.1974	2.8896	1.2924	-0.1356	0.1439	1.1195
**C6**	5.4903	4.6656	0.5175	2.0624	0.3799	2.5398	0.9984	-0.4580	-0.5103	-0.7268
**C7**	5.6640	4.9655	2.0657	0.4489	-0.7050	2.0711	0.6639	-0.6712	-0.3362	-0.3168
**C8**	5.5771	4.8142	-1.5829	1.2619	-0.8464	2.3161	0.8303	0.2265	-0.3135	0.8250
**C9**	5.5291	4.7318	0.7763	2.0238	-0.1832	2.1829	0.7375	0.0540	0.5222	-0.6796
**C10**	5.4721	4.6347	0.7634	1.8828	-0.7121	2.8377	1.2464	0.2472	-0.5897	0.9152
**C11**	5.4245	4.5545	-0.8384	0.8208	-1.7827	2.7056	1.1331	0.1478	-0.0549	-1.0527
**C12**	5.4414	4.5830	1.0225	-1.8759	0.1356	2.6893	1.1194	-0.2525	0.7593	0.6922
**C13**	5.5280	4.7298	-1.7554	-0.9651	0.8468	2.8140	1.2257	0.5441	-0.0466	-0.9631

Table S3. Comparison of CP43 chlorophyll site energies from the literature. All values are in eV.

Site	Muh et al.[22] [a]	Muh et al.[22] [b]	Shibata et al.[23] [c]	Shibata et al.[23] [d]	Sarngadharan et al.[24]	Saito et al.[25]	QM/MM (this work)	PMM (this work)
**C1**	1.839	1.843	1.853	1.842	1.818	2.127	1.846	1.873
**C2**	1.851	1.857	1.860	1.863	1.831	2.154	1.867	1.894
**C3**	1.843	1.840	1.853	1.862	1.817	2.114	1.861	1.867
**C4**	1.845	1.843	1.831	1.829	1.866	2.142	1.928	1.945
**C5**	1.846	1.847	1.853	1.834	1.842	2.152	1.938	1.915
**C6**	1.861	1.859	1.853	1.855	1.851	2.129	1.970	1.963
**C7**	1.839	1.838	1.833	1.841	1.842	2.122	1.885	1.890
**C8**	1.834	1.832	1.846	1.856	1.841	2.122	1.915	1.895
**C9**	1.828	1.834	1.825	1.818	1.848	2.096	1.881	1.912
**C10**	1.836	1.835	1.884	1.902	1.845	2.115	1.955	1.942
**C11**	1.823	1.823	1.862	1.830	1.831	2.136	1.986	1.964
**C12**	1.833	1.839	1.849	1.842	1.839	2.110	1.926	1.925
**C13**	1.861	1.858	1.873	1.870	1.865	2.142	1.961	1.937

[a] Based on 3ARC[1]
[b] Based on 4IL6[26]
[c] Fit of linear optical spectra
[d] Fit of absorption and LD spectra

Table S4. Excitonic coupling constants (upper panel) and corresponding standard deviations (lower panel) between CP43 chlorophylls computed using the **MD-PMM** approach. All values are reported in cm^{-1}.

Chl	C1	C2	C3	C4	C5	C6	C7	C8	C9	C10	C11	C12	C13
**C1**		10.89	21.90	3.09	-3.07	-12.22	21.05	7.15	10.89	2.70	-2.52	0.63	-0.11
**C2**	6.05		8.14	154.07	-8.67	-16.15	-1.01	16.94	23.26	73.09	5.05	12.33	-10.27
**C3**	5.31	7.89		-36.43	4.48	-0.45	7.88	-4.39	-11.26	23.04	8.19	25.02	5.71
**C4**	1.07	8.80	3.02		-3.40	4.57	-2.08	-1.53	-0.97	-34.68	-4.46	-4.72	5.09
**C5**	0.86	1.08	0.36	0.48		25.46	-98.32	-5.90	-31.38	-1.18	-0.81	-1.55	5.03
**C6**	1.38	0.72	0.64	0.20	1.88		-77.69	-1.14	-32.26	-12.14	-4.85	-4.15	10.23
**C7**	1.53	1.89	0.98	0.42	5.26	15.33		17.84	-112.59	-4.61	-9.06	-34.46	26.34
**C8**	0.25	0.87	0.47	0.81	3.34	0.54	2.23		130.37	66.94	1.78	2.11	-5.16
**C9**	0.71	1.85	1.11	0.86	1.45	1.32	10.64	10.68		16.20	-117.64	36.43	-6.15
**C10**	1.12	7.47	2.36	3.33	1.13	0.46	1.95	4.51	12.58		-97.23	19.40	-11.76
**C11**	0.34	0.73	0.72	0.67	0.42	0.26	0.64	4.72	5.37	5.87		25.24	-13.91
**C12**	0.79	1.40	2.18	0.46	0.66	1.56	5.37	0.90	6.66	1.77	1.07		-48.50
**C13**	0.57	0.38	1.15	0.18	0.26	0.45	0.87	0.41	1.72	0.74	1.11	9.71	

Table S5. Relative center-to-center distances (in Å) of the 13 CP43 Chls, $Chlz_{D1}$ and D1-RC pigments based on the 3WU2 crystal structure.[1] Distances less than 12 Å are shown in red, 12–15 Å are shown in pink. In CP43, C5 is closest the D1 pigments ($Chlz_{D1}$, $Pheo_{D1}$ and Chl_{D1}) while C4 is closer to P_{D1} (distances in green).

	C1	C2	C3	C4	C5	C6	C7	C8	C9	C10	C11	C12	C13	$Chlz_{D1}$
**C2**	**13.1**													
**C3**	**12.7**	**12.1**												
**C4**	22.0	**11.2**	16.3											
**C5**	23.9	20.9	29.3	30.5										
**C6**	16.9	24.1	25.3	35.1	16.9									
**C7**	18.8	18.9	22.1	29.4	**12.1**	**8.7**								
**C8**	26.5	17.5	24.9	23.6	**14.6**	24.1	15.5							
**C9**	22.5	16.4	19.1	23.6	17.9	19.9	**11.7**	**9.1**						
**C10**	20.8	**10.2**	15.1	**14.3**	21.6	25.8	18.3	**11.4**	**9.6**					
**C11**	31.2	21.9	24.5	24.4	25.9	30.5	22.2	**11.9**	**10.7**	**11.8**				
**C12**	20.0	20.7	16.4	28.6	24.7	16.7	**13.3**	20.8	**12.0**	17.5	20.0			
**C13**	28.0	26.0	19.9	30.6	32.8	26.8	22.5	24.7	16.1	19.9	18.3	**10.4**		
**$Chlz_{D1}$**	33.7	34.3	43.7	42.4	**21.9**	31.2	31.4	34.9	38.9	39.8	46.6	43.8	53.0	
**Chl_{D1}**	41.0	33.3	45.4	35.4	**28.5**	44.0	39.2	31.9	39.6	36.3	41.8	49.1	55.1	24.7
**$Pheo_{D1}$**	39.0	30.8	42.6	34.7	**21.5**	38.3	32.7	24.4	32.7	31.4	34.7	43.0	48.7	24.3
**P_{D1}**	38.9	29.1	40.8	**28.1**	30.6	45.1	39.3	29.7	37.0	31.6	37.4	46.8	51.3	32.0

Table S6. QM/MM and TD-DFT (ωB97X-D3(BJ)/def2-TZVP) computed excitation energies (in eV) and corresponding oscillator strengths and NTO analysis for selected chlorophyll pairs in CP43. The geometries in all cases are derived from QM/MM optimizations of the pigments embedded inside the complete PSII monomer for snapshot 1. (LE: local excitations, CT: Charge Transfer states).

Pair	Mg-Mg (Å)	State	Es (eV)	f_{osc}	NTO character	weight
**C12-C13**	10.1	1	1.920	0.21	LE (C12)	0.84
					LE (C12)	0.19
		2	1.967	0.24	LE (C13)	0.83
					LE (C13)	0.19
		3	2.410	0.06	LE (C12)	0.77
					LE (C12)	0.24
		4	2.46	0.15	LE (C13)	0.81
					LE (C13)	0.19
		5	3.222	0.12	delocalized exciton + CT	0.88
**C2-C10**	10.4	1	1.843	0.13	LE(C2)	0.84
					LE(C2)	0.20
		2	1.943	0.26	**LE (C10) + CT (C2 → C10)**	**0.86**
					LE (C10)	0.10
		3	2.001	0.07	**LE (C10) + CT (C2 → C10)**	**0.89**
					LE (C10)	0.10
		4	2.255	0.03	LE(C2)	0.69
					LE(C2)	0.34
		5	2.465	0.17	LE (C10)	0.83
					LE (C10)	0.19
**C6-C7**	9.0	1	1.885	0.13	LE(C7)	0.84
					LE(C7)	0.20
		2	1.963	0.26	LE (C6)	0.84
					LE (C6)	0.19
		3	2.384	0.07	LE(C7)	0.74
					LE(C7)	0.28
		4	2.453	0.03	LE (C6)	0.79
					LE (C6)	0.21
		5	3.218	0.17	delocalized exciton + CT	0.80
**C8-C9**	9.5	1	1.882	0.23	LE(C9)	0.82
					LE(C9)	0.20
		2	1.913	0.24	LE (C8)	0.83
					LE (C8)	0.18
		3	2.352	0.04	LE(C9)	0.77
					LE(C9)	0.25
		4	2.441	0.06	LE (C8)	0.75
					LE (C8)	0.26
		5	3.251	0.33	LE(C9)	0.67
					LE(C9)	0.27
**C9-C10**	9.3	1	1.888	0.17	LE(C9)	0.83
					LE(C9)	0.20
		2	1.942	0.25	LE(C10)	0.84
					LE(C10)	0.18
		3	2.365	0.04	LE(C9)	0.76
					LE(C9)	0.26
		4	2.433	0.11	LE(C10)	0.81
					LE(C10)	0.21
		5	3.094	0.00	CT (C10 → C9)	1.00
**C7-C9**	11.4	1	1.868	0.39	LE (C9)	0.44
					LE (C7)	0.41

		2	1.891	0.09	LE (C7)	0.43
					LE (C9)	0.40
		3	2.350	0.07	LE (C9)	0.73
					LE (C9)	0.24
		4	2.37	0.04	LE (C7)	0.69
					LE (C7)	0.28
		5	3.278	0.30	LE (C9)	0.44
					LE (C7)	0.28
**C9-C11**	11.0	1	1.864	0.30	LE (C9)	0.83
					LE (C9)	0.20
		2	1.981	0.20	LE (C11)	0.83
					LE (C11)	0.18
		3	2.329	0.07	LE (C9)	0.76
					LE (C9)	0.27
		4	2.466	0.14	LE (C11)	0.83
					LE (C11)	0.19
		5	3.071	0.00	CT (C9 $\rightarrow$ C11)	1.00
**C2-C4**	11.8	1	1.868	0.32	LE(C2)	0.75
					LE(C2)	0.20
		2	1.899	0.098	LE(C4)	0.73
					LE(C4)	0.22
		3	2.291	0.02	LE(C2)	0.69
					LE(C2)	0.34
		4	2.320	0.00	LE(C4)	0.63
					LE(C4)	0.40
		5	3.166	0.64	LE(C4)	0.61
					LE(C4)	0.42
**C8-C10**	11.2	1	1.899	0.23	LE(C8)	0.80
					LE(C8)	0.17
		2	1.950	0.24	LE(C10)	0.80
					LE(C10)	0.17
		3	2.439	0.16	LE(C8)	0.62
					LE(C8)	0.22
		4	2.445	0.04	LE(C10)	0.67
					LE(C10)	0.18
		5	3.127	0.00	CT (C10 $\rightarrow$ C8)	1.00
**C10-C11**	11.9	1	1.958	0.34	LE(C10)	0.80
					LE(C10)	0.17
		2	1.994	0.18	LE(C11)	0.79
					LE(C11)	0.17
		3	2.451	0.26	LE(C10)	0.75
					LE(C10)	0.17
		4	2.484	0.11	LE(C11)	0.76
					LE(C11)	0.16
		5	2.546	0.00	CT (C10 $\rightarrow$ C11)	1.00
**C5-C7**	12.1	1	1.882	0.35	LE(C7)	0.78
					LE(C7)	0.18
		2	1.933	0.17	LE(C5)	0.79
					LE(C5)	0.16
		3	2.392	0.05	LE(C7)	0.72
					LE(C7)	0.30
		4	2.440	0.13	LE(C5)	0.83
					LE(C5)	0.19
		5	3.331	0.50	LE(C7)	0.64
					LE(C7)	0.34

Table S7. QM/MM and TD-DFT (ωB97X-D3(BJ)/def2-TZVP) computed excitation energies (in eV) and corresponding oscillator strengths and NTO analysis for the C2-C10 pair in CP43. The geometries in all cases are derived from QM/MM optimizations of the pigments embedded inside the complete PSII monomer in snapshots 2-10 (LE: local excitations, CT: Charge Transfer states).

Snapshot	Mg-Mg (Å)	State	Es (eV)	f_{osc}	NTO character	weights
**2**	10.6	1	1.875	0.20	LE(C2)	0.83
					LE(C2)	0.22
		2	1.967	0.26	LE (C10)	0.85
					LE (C10)	0.18
		3	2.230	0.00	**CT (C2 → C10)**	**0.98**
		4	2.320	0.04	LE(C2)	0.69
					LE(C2)	0.34
		5	2.478	0.15	LE (C10)	0.80
					LE (C10)	0.21
**3**	10.3	1	1.838	0.12	**LE (C2) + CT (C2 → C10)**	**0.87**
					LE(C2)	0.17
		2	1.901	0.10	**LE (C2) + CT (C2 → C10)**	**0.94**
					LE(C2)	0.06
		3	1.998	0.23	LE (C10)	0.85
					LE (C10)	0.18
		4	2.273	0.02	LE(C2)	0.68
					LE(C2)	0.35
		5	2.447	0.01	**CT (C2 → C10)**	**0.98**
**4**	10.3	1	1.868	0.18	LE(C2)	0.83
					LE(C2)	0.22
		2	1.979	0.27	LE (C10)	0.85
					LE (C10)	0.17
		3	2.099	0.00	**CT (C2 → C10)**	**0.99**
		4	2.298	0.04	LE(C2)	0.69
					LE(C2)	0.34
		5	2.491	0.18	LE (C10)	0.81
					LE (C10)	0.20
**5**	10.1	1	1.869	0.18	LE(C2)	0.83
					LE(C2)	0.22
		2	1.953	0.28	LE (C10)	0.85
					LE (C10)	0.18
		3	2.237	0.00	**CT (C2 → C10)**	**0.98**
		4	2.279	0.04	LE(C2)	0.69
					LE(C2)	0.34
		5	2.461	0.14	LE (C10)	0.80
					LE (C10)	0.22
**6**	10.1	1	1.855	0.19	LE(C2)	0.83
					LE(C2)	0.22
		2	1.981	0.29	LE (C10)	0.85
					LE (C10)	0.17
		3	2.237	0.00	**CT (C2 → C10)**	**0.90**
		4	2.288	0.03	LE(C2)	0.58
					LE(C2)	0.44

		5	2.509	0.17	LE (C10)	0.81
					LE (C10)	0.21
**7**	10.2	1	1.842	0.18	LE(C2)	0.84
					LE(C2)	0.21
		2	1.966	0.15	**LE (C10) + CT (C2 → C10)**	**0.92**
					LE (C10)	0.10
		3	2.018	0.14	**LE (C10) + CT (C2 → C10)**	**0.88**
					LE (C10)	0.12
		4	2.271	0.04	LE(C2)	0.69
					LE(C2)	0.34
		5	2.509	0.14	LE (C10)	0.87
					LE (C10)	0.14
**8**	10.4	1	1.859	0.17	LE(C2)	0.83
					LE(C2)	0.21
		2	1.939	0.31	LE (C10)	0.85
					LE (C10)	0.17
		3	2.102	0.00	**CT (C2 → C10)**	**0.99**
		4	2.282	0.04	LE(C2)	0.70
					LE(C2)	0.33
		5	2.461	0.15	LE (C10)	0.81
					LE (C10)	0.20
**9**	9.9	1	1.803	0.11	**LE (C2) + CT (C2 → C10)**	**0.88**
					LE(C2)	0.15
		2	1.895	0.13	**LE (C2) + CT (C2 → C10)**	**0.91**
					LE(C2)	0.10
		3	1.955	0.23	LE (C10)	0.86
					LE (C10)	0.17
		4	2.240	0.03	LE(C2)	0.69
					LE(C2)	0.34
		5	2.440	0.05	**LE (C10) + CT (C2 → C10)**	**0.94**
**10**	10.5	1	1.856	0.18	LE(C2)	0.83
					LE(C2)	0.23
		2	1.923	0.29	LE (C10)	0.85
					LE (C10)	0.18
		3	2.257	0.00	**LE (C2) + CT (C2 → C10)**	**0.59**
					LE (C10)	0.42
		4	2.309	0.04	**LE (C2) + CT (C2 → C10)**	**0.76**
					LE(C2)	0.24
		5	2.436	0.13	LE (C10)	0.80
					LE (C10)	0.22

Snapshot 2 (5 ns), S3

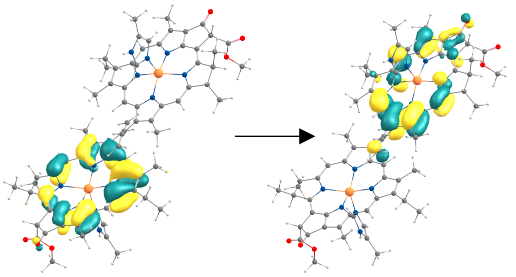

Snapshot 3 (10 ns), S1 and S2

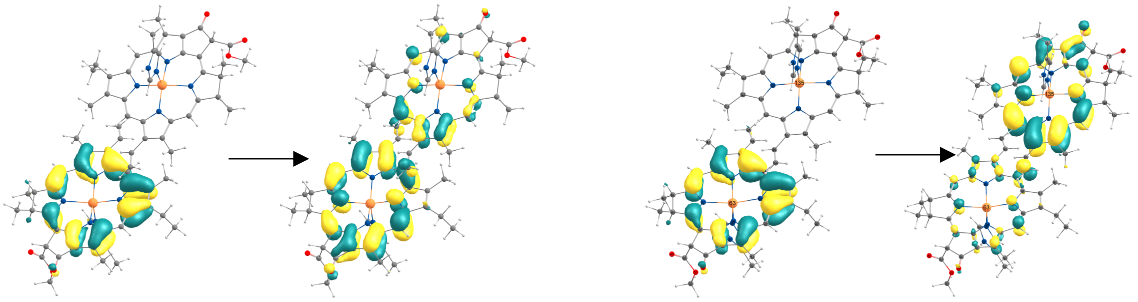

Snapshot 4 (15 ns), S3

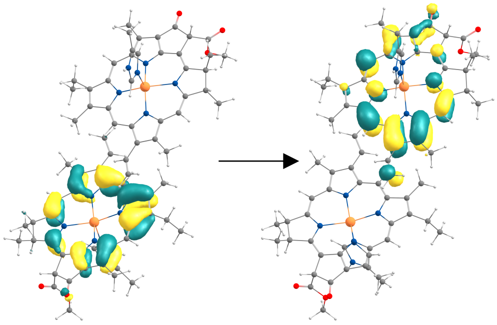

Snapshot 5 (20 ns), S3

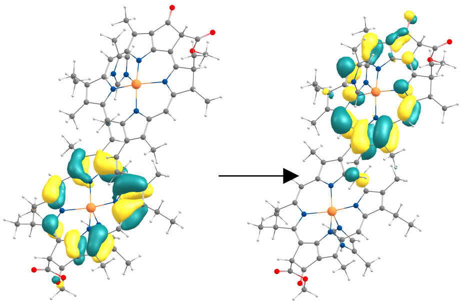

Snapshot 6 (25 ns), S3

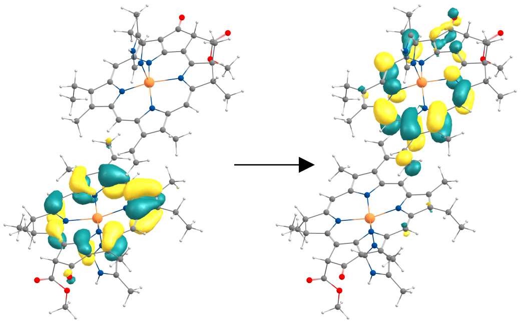

Figure S5. Natural Transition Orbitals (NTOs) for the lowest excited state with significant CT character for the C2-C10 dimer in snapshots 2-10.

Snapshot 7 (30 ns), S2

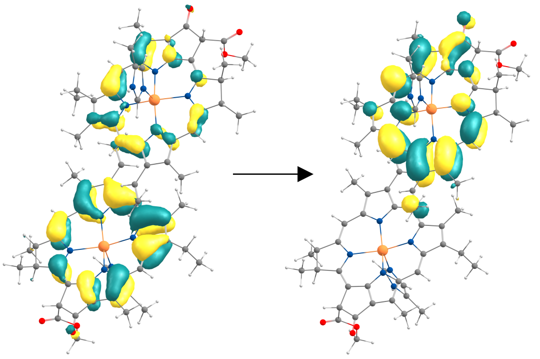

Snapshot 8 (35 ns), S3

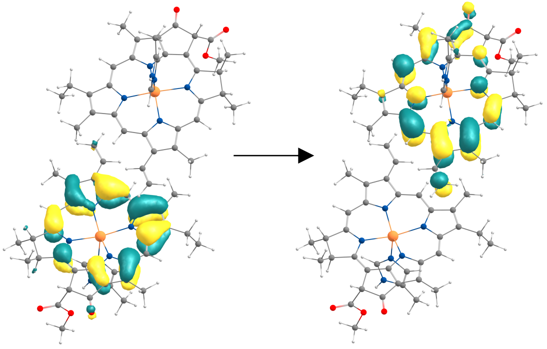

Snapshot 9 (40 ns), S1 and S2

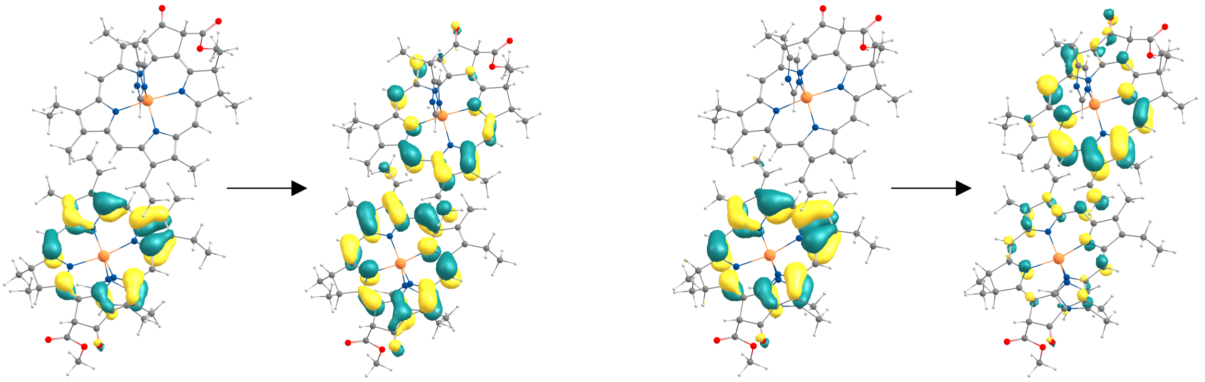

Snapshot 10 (45ns), S3

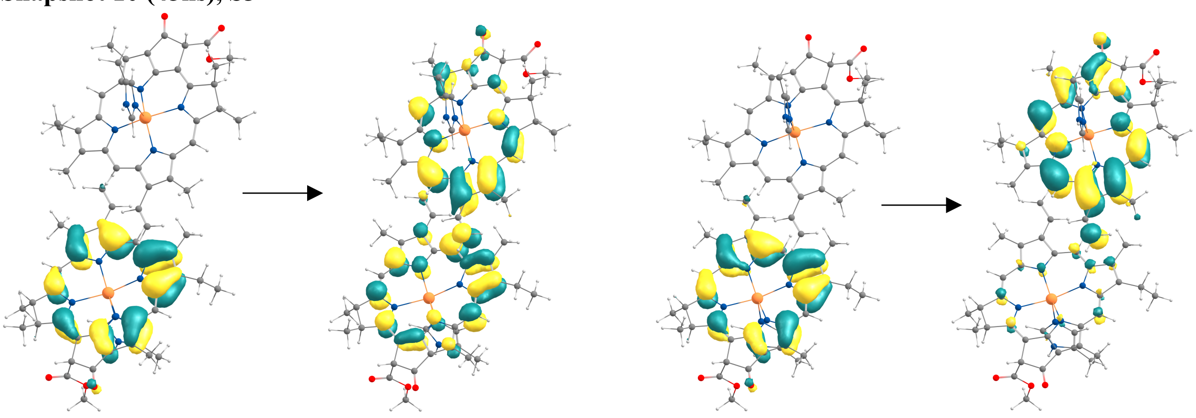

Figure S5 (continued). Natural Transition Orbitals (NTOs) for the lowest excited state with significant CT character for the C2-C10 dimer in snapshots 2-10.

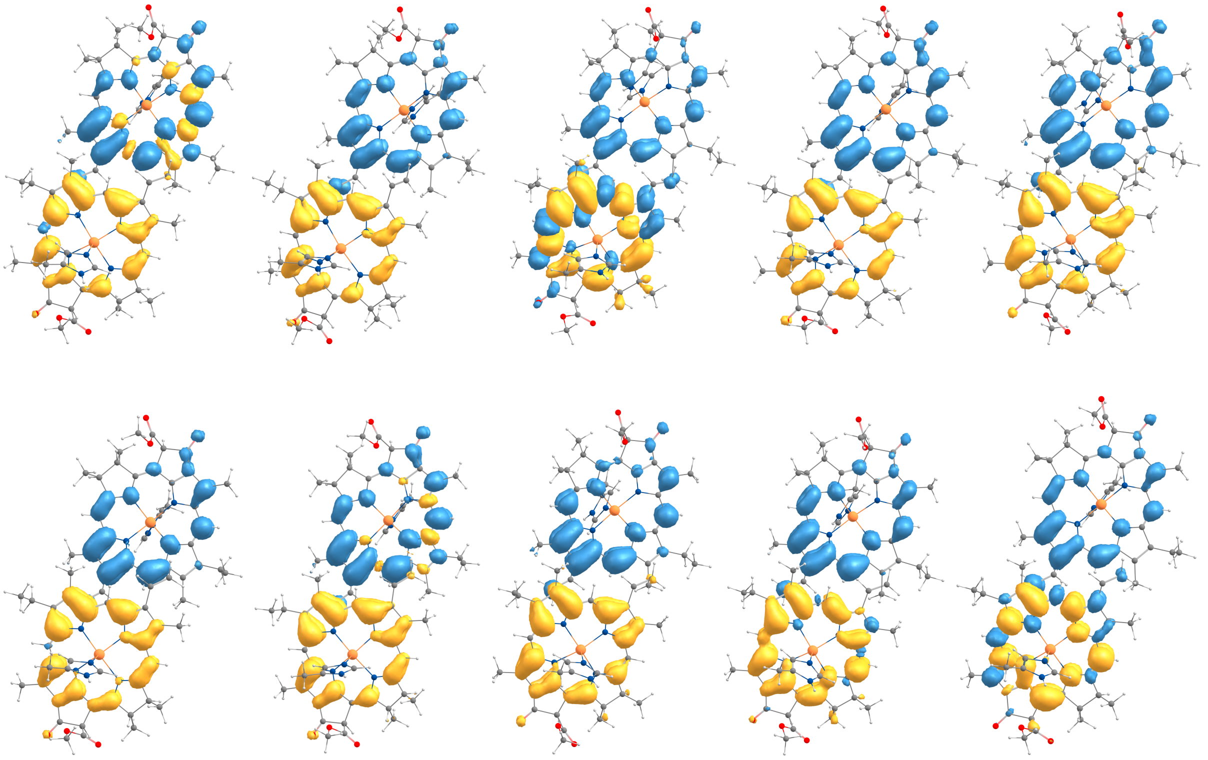

Figure S6. TD-DFT difference densities for the lowest excited states with CT character for the C2-C10 dimer in the 'crystal-like' (snapshot 1) and snapshots 2-10.

Table S8. Comparison of different range-separated functionals to compute the Chl a ionization potential (IP) and electron affinity (EA), the energy of the Highest Occupied Molecular Orbital (HOMO) for Chl and Chl^{-}.[27] All energy values are in eV. HFX is the range of exact (Hartree–Fock) exchange included in each functional, and ω (in $Bohr^{-1}$) is the parameter controlling the range separation.

DFT Method	HFX	ω	HOMO (Chl)	IP (Chl^{+}/Chl)	HOMO (Chl^{-})	EA (Chl/Chl^{-})	CT state energy
CAM-B3LYP[28]	19–65%	0.33	-6.1184	6.2733	-1.7153	1.9350	1.302
LC-BLYP[29]	0–100%	0.33	-6.8345	6.2740	-2.5112	2.0611	1.930
ωB97[30]	0–100%	0.40	-6.8535	6.0935	-2.5049	2.0602	2.104
ωB97X-V[31]	16.7–100%	0.30	-6.7753	6.2733	-2.6160	1.9738	1.943
ωB2PLYP[32]	53–100%	0.30	-6.5902	6.5702	-2.4626	1.7388	--
LC-PBE[33]	0–100%	0.47	-7.1880	6.3005	-3.0015	2.3925	1.958

Example ORCA input file for the QM/MM TD-DFT calculations conducted in the present work

```
! QMMM NoTrah
! wB97X-D3BJ RIJCOSX verytightSCF

%tddft
 TDA false
 nroots 8
 maxdim 20
 donto true
 NTOThresh 1e-4
end

%pal nprocs 8 end
%maxcore 16000

%basis
 Basis "Def2-TZVP"
 AuxJ  "Def2/J"
 AuxC  "Def2-TZVP/C"
 AuxJK "Def2/JK"
end

%qmmm
 ORCAFFFilename "ps2-A1-final.ORCAFF.prms"
 Embedding Electrostatic
 ChargeAlteration CS
 QMAtoms end
end

*pdbfile 0 1 chl-opt.pdb
```

Note: complete ORCA output files of all TD-DFT calculations are provided in a separate supporting data set hosted by the Open Research Data Repository of the Max Planck Society at: https://doi.org/10.17617/3.S2DPUE

Cartesian coordinates (in Å) of QM regions from the QM/MM geometry optimizations of the 13 CP43 chlorophylls and of Chl_{ZD1}

Note: These coordinates are also provided as a separate plain-text file.

Note: PDB files that contain the optimized QM structures within the complete MM setup of the PSII monomer, as well as complete force field parameter files to enable reproduction of the complete simulations, are provided in a separate supporting data set, hosted by the Open Research Data Repository of the Max Planck Society at: https://doi.org/10.17617/3.S2DPUE

To facilitate use of these PDB files, the list below indicates the correspondence between the Chl labels used in this work and the residue numbers in the PDB files.

Site	Chl in PDB	Ligand in PDB
**C1**	1146	H563
**C2**	1147	H756
**C3**	1148	H444
**C4**	2736	
**C5**	1149	H767
**C6**	1150	H577
**C7**	2737	
**C8**	1151	H770
**C9**	1152	H379
**C10**	1153	H382
**C11**	2738	N365
**C12**	1154	H490
**C13**	1155	H458
**Chl_{ZD1}**	1145	H118

```
C1
88
C   109.942118107884     63.430277071805     84.822841610029
H   110.322679352993     63.116007714856     85.804607033856
H   110.804500421759     63.627637971882     84.172264450002
C   109.101613289299     64.661100257664     84.890923304930
N   108.250592035459     64.958301115770     85.939133238347
H   108.073006836008     64.357949026038     86.748335579086
C   107.634384261784     66.136312757261     85.677126360575
H   106.931717627102     66.619112486604     86.348183605036
N   108.034263990189     66.611440268213     84.507586647159
C   108.950608015071     65.708943093134     84.009156337919
H   109.444867511205     65.882903568564     83.058714690414
C   105.347785579216     72.189962612159     86.868265664590
C   103.733718163019     65.817335356744     80.216258965993
C   111.308839955818     68.698235780702     79.332540123928
C   110.292131579939     71.451380096640     86.889078934188
N   106.295464378594     69.379503129451     85.136102569923
C   104.022687090885     65.866345641133     78.900884518309
C   111.161269572482     70.143228894689     78.837531947464
C   108.793026019970     71.267102581316     87.390381634199
```

N	105.706267861219	67.858564116940	82.627807203343
O	111.143644262299	72.102576682389	87.474145483635
C	108.823929640112	70.451266203735	88.679818728651
N	109.096204941100	69.520082792793	84.166025411821
C	109.417716838471	70.557997018899	90.980244798628
C	108.100838096093	70.515960790211	86.263749417908
C	104.189542310911	68.301180287148	84.515857558075
C	106.678729088412	67.103593682265	80.511397939647
C	110.691168098837	69.186622580913	82.361439645729
C	104.233148246463	68.614106395636	87.675746856187
C	102.249491435827	66.442519363344	82.870353190403
C	108.755627213884	67.118098628373	78.178270684389
C	112.640148222905	70.808821812397	84.345432392646
C	106.755967133968	70.197114253492	86.135174169240
C	104.512321207117	67.710584638169	83.276667946663
C	107.991821541485	67.598299202693	80.635000914229
C	110.378174396038	69.642536292142	83.648780380073
O	108.589101563829	69.256449223241	88.740097714047
C	105.622682083308	70.680145375268	87.020193814580
C	103.623470833292	66.885403132014	82.483441415897
C	108.972497173789	67.621066498467	79.566926360526
C	111.228936986821	70.382946508984	84.575249122971
O	109.184116643763	71.225515011409	89.718145159365
C	104.473607205384	69.714274589920	86.632879262690
C	104.296713168305	66.595474333931	81.300604630375
C	110.094903706703	68.245133229688	80.076813041149
C	110.390228775243	70.704076847663	85.643635247253
C	104.978793047490	69.108243365295	85.331797676717
C	105.622429403659	67.203704363600	81.422980661596
C	109.801563585587	68.565181255403	81.463841962917
C	109.102472896176	70.168664653417	85.335030246205
N	108.516371449011	68.171196840295	81.762227207103
Mg	107.466715523073	68.436950725918	83.559843386837
H	106.260662094161	72.747491247714	87.108062612362
H	104.578196292601	72.519744502759	87.578772241207
H	102.946738707870	65.116177668923	80.522659929958
H	111.507875051838	68.025399001892	78.485351820530
H	112.184189746108	68.661572789765	79.998810123747
H	104.748033736692	66.568615655774	78.491573642369
H	103.521598687603	65.200573830688	78.198380705427
H	111.030952694655	70.803549999722	79.704034144403
H	110.276675501309	70.258355576228	78.195969657190
H	112.049275960258	70.475335951168	78.277439327391
H	108.391217816758	72.266859080293	87.614080280230
H	110.500757393525	70.439410784982	91.114847825893
H	108.922752406407	69.580713959110	90.996802042183
H	109.026285192080	71.216569146599	91.760984056157
H	103.172289580094	68.132585810947	84.874480697068
H	106.461486715780	66.587066169244	79.577899600782
H	111.705888982572	69.370901063886	82.003092684536
H	103.829625273773	69.036087606376	88.605164886372
H	105.169734257727	68.086261118070	87.911397131907
H	103.505379951294	67.879765389362	87.304702915456
H	102.119153677149	66.438742807903	83.960631582615
H	102.042085779085	65.422085212626	82.514631177958
H	101.462208172697	67.084382072425	82.441962881486
H	109.717791462217	66.976632735348	77.669716044930
H	108.147717799763	67.816390610855	77.577921295814

H	108.230733860005	66.150238842843	78.172502672158
H	113.224957273718	70.045279921901	83.811755630332
H	113.137628781757	71.039550307505	85.296196034917
H	112.664685047832	71.727808709819	83.735998244179
H	105.904374075608	70.522890104011	88.073427243833
H	103.524518732935	70.249027269323	86.483927779333
O	111.134884149960	74.175737939602	89.542747143064
H	111.984206170065	74.654646201668	89.485901327557
H	111.132019455426	73.549341469669	88.791083786571
H	109.353647105734	62.592315497268	84.397766716665
H	105.044292548816	72.563432777818	85.862592706228

C2

101

C	104.198845310509	76.223534278193	92.770829455742
H	103.270897577928	75.822743362857	93.199356512176
H	104.283925482458	77.278827620695	93.066655190295
C	104.151677147447	76.078199913746	91.271178653396
C	104.929129848303	76.874623952010	90.419196528843
H	105.591191431235	77.627526796479	90.847812018336
C	104.855069531952	76.748894270171	89.031468556578
H	105.464931258332	77.386383496160	88.386825199218
C	103.993284464617	75.800506255114	88.459631339715
O	103.860714871392	75.635114991498	87.106372490455
H	104.714359019004	75.869118207760	86.676293991394
C	103.206319006636	74.999914609977	89.294765880370
H	102.562340260859	74.251539309387	88.833515142770
C	103.285072653223	75.147615443075	90.678635555311
H	102.654920054835	74.528055329416	91.320701434815
C	99.764159298549	81.172105365391	82.673045782781
H	99.413426276764	81.146353993573	83.711017774299
H	99.469191939096	80.238572613223	82.177436575913
C	101.238873336170	81.377196727407	82.524221462910
N	102.000302787158	82.156296468401	83.373639671780
H	101.631750308683	82.598684309964	84.219584889718
C	103.224125955700	82.336531718586	82.817704329114
H	104.020121383308	82.908878609322	83.282126038355
N	103.298605190111	81.702813255117	81.651830553106
C	102.068918899790	81.100254725628	81.462304988033
H	101.862904408535	80.503735176036	80.579076675816
C	108.681254409124	78.892345577814	83.690329038377
C	106.127560398117	85.803599439210	77.518501102113
C	101.414815465675	79.555155110525	75.981659465428
C	104.430961683970	76.633606936166	83.039843650114
N	106.410348054554	80.874281061551	81.864416377175
C	106.813518837207	86.955945133920	77.380878730208
C	99.981326929289	80.043322535884	76.248424647387
C	105.357520331519	77.687957620127	83.760286406594
N	105.916806560523	82.759364885909	79.600619905367
O	104.131504124868	75.543894491726	83.500261079783
C	104.720359164240	78.336546985177	84.986984118757
N	104.364677193919	79.160693250034	80.538401874323
C	102.666001583012	78.940781487036	85.990616814419
C	105.567008026770	78.797107312115	82.731695119055
C	107.480106545979	83.034881747291	81.488198600002
C	104.682076913594	83.086772291976	77.503934022036
C	102.916295764225	78.692522517102	78.637593503640

C	108.028511754229	82.432158265103	84.531514047537
C	107.867191476582	85.878569246850	80.266471284547
C	102.604673805306	82.417773738813	75.248290183522
C	102.607880683244	75.867429110933	80.094838491846
C	106.468800680362	79.853557680100	82.773782661709
C	106.820774155478	83.490978364674	80.326849097306
C	103.919754999681	81.904823321746	77.456815550760
C	103.515127008070	78.312770036689	79.846117827234
O	105.344745152210	78.900225081064	85.865575396400
C	107.629541798353	80.021719984797	83.739498561057
C	107.037608354204	84.779437753028	79.698039721286
C	102.970117338918	81.563044447012	76.414865415704
C	103.333543539074	77.071589714638	80.583309072126
O	103.374776679622	78.283945639759	84.920999615299
C	108.165757811464	81.433531223651	83.379345203484
C	106.275886126961	84.783092153637	78.531951981572
C	102.460934599649	80.320341937840	76.734823823753
C	104.046607328913	77.245958858873	81.767257909726
C	107.336170588496	81.820992841787	82.160727010299
C	105.573989772696	83.501406638319	78.496909218958
C	103.108908671542	79.916923048541	77.973541050933
C	104.673407068801	78.525508536622	81.674531414136
N	103.966625132523	80.910606648571	78.395609838926
Mg	104.890960376983	81.118604886675	80.305283518789
H	108.288456021201	77.982597538835	84.160353644029
H	109.572055365405	79.182956054588	84.260662945155
H	105.360963182963	85.597978132860	76.764015390105
H	101.480397677508	78.484350485721	76.229168821684
H	101.615538277399	79.637261671159	74.898748821990
H	107.605565532275	87.250466990561	78.063611414791
H	106.596773249544	87.627262601133	76.553616720324
H	99.243078440428	79.407403322319	75.738408762542
H	99.845572588866	81.072343249274	75.888156738916
H	99.758176615427	80.026060497410	77.324800535688
H	106.280997208749	77.208762674154	84.101407416370
H	101.689404054729	78.453540315241	86.051054091385
H	102.552491893080	80.006727054142	85.742258048302
H	103.214433710061	78.831644506711	86.933238842498
H	108.210655991824	83.724792184780	81.912668647218
H	104.540185680698	83.770157423893	76.664977305028
H	102.228365711174	77.982413064320	78.174417101426
H	106.972900039782	82.537494244899	84.817758033458
H	108.403862643936	83.426577978837	84.254551751001
H	108.592918680393	82.089020884008	85.409748620638
H	107.414763846899	86.856919028357	80.057827526768
H	108.889040956746	85.902358584900	79.855653586231
H	107.962780522893	85.786827583689	81.353771408584
H	103.492064485845	82.795113858600	74.721555574797
H	102.030478890700	83.304590444275	75.554560749578
H	101.980149898926	81.865497804670	74.534274662825
H	101.629292878270	75.749456091111	80.580294766120
H	103.187159605010	74.961045031080	80.318575080227
H	102.449784942086	75.921187120577	79.008466166909
H	107.232453556377	80.018913213212	84.767073777947
H	109.230856532604	81.374991507337	83.099758236467
H	105.039726044601	75.660785399070	93.265297733037
H	99.288278876883	82.026215142491	82.147707598774
H	109.003699772400	78.623900779050	82.657104603431

C3
85

C	119.794613278974	81.921605780046	80.369494600604
H	119.969225917318	82.507915785777	81.281988905288
H	119.113045803101	82.488599363291	79.726643198077
C	119.172943803651	80.604030554297	80.681791631654
N	119.429827155271	79.908144495743	81.847109087393
H	119.987339839827	80.281949648315	82.621609851784
C	118.719762424703	78.752627837949	81.824976261967
H	118.724713812814	78.015181239948	82.621007572686
N	118.025397228004	78.657980601955	80.697041416870
C	118.292837539379	79.806225979800	79.981614129528
H	117.815438300916	79.984797871017	79.023904674269
C	114.472694018687	76.109078351616	85.276574598668
C	120.296750760383	73.290251969132	78.026002657608
C	114.639843086285	77.900960209881	74.826024682234
C	112.787766069530	79.684608393874	82.568698208363
N	116.397214497171	76.490699700570	82.256125139549
C	120.963038205554	72.119669512245	78.104447979392
C	113.406621778246	77.010849808510	74.616556449458
C	113.583848590985	78.771672915336	83.586539565508
N	117.964040829393	75.430735798578	79.936469576813
O	111.961262334845	80.518607833565	82.890189756145
C	114.281358509202	79.640118215840	84.611263803243
N	114.913510053727	78.069959915202	80.221012388025
C	115.911182494790	81.322427782957	84.842370625484
C	114.567324122254	77.992264481578	82.723017843725
C	118.219265003452	74.862482703652	82.324432173962
C	118.129494245160	75.415694003230	77.484416390313
C	114.424688264714	78.553316923184	77.891551874302
C	118.060014271977	75.802504158215	85.325328710040
C	120.335570269135	72.836514583442	81.216237759046
C	117.113542287319	75.869785002454	74.564092465565
C	112.157790106446	80.463715013876	79.239006889822
C	115.518575554049	77.069494182984	83.129773085215
C	118.540127057729	74.719986667053	80.955617674273
C	117.102675284044	76.312202834039	77.144680296782
C	114.183041402398	78.749591263316	79.254378667430
O	114.035193896786	79.691514097743	85.795854610227
C	115.732227507370	76.588650639171	84.549292531841
C	119.496942407190	73.775273477421	80.421520356023
C	116.602537304610	76.535298083221	75.801360124525
C	113.151209511069	79.569563924767	79.887386117365
O	115.242841835273	80.374909622747	83.992267574066
C	116.872993101185	75.551285642125	84.396335357944
C	119.495654177552	73.945256800484	79.038818222920
C	115.541512217643	77.411914223568	75.916714906666
C	113.293147587956	79.330633966893	81.244589642633
C	117.214843049596	75.627587053074	82.912977286764
C	118.517178545294	74.990560507782	78.758519548084
C	115.411633006979	77.719138744315	77.332827357880
C	114.357901185163	78.396868677219	81.393643383808
N	116.378657801921	77.046617359842	78.047174853239
Mg	116.677856259835	77.038424984074	80.148594261419
H	113.820114301984	76.958102003977	85.511514840590
H	114.745540922241	75.672277115646	86.242984366158

H	120.364902769382	73.829852459197	77.074539834761
H	115.207567588805	77.966594383266	73.882102497811
H	114.306177517013	78.927442919605	75.047994492977
H	120.932784509845	71.479059024256	78.984819852998
H	121.547961688902	71.761696142076	77.256179941248
H	112.805762675060	76.963927129138	75.535737114273
H	113.696963961120	75.981763826067	74.359916282088
H	112.769090238501	77.404069186176	73.810140389745
H	112.852718150518	78.147819498233	84.114683200049
H	116.739090025094	81.724492138039	84.250465180875
H	116.268986531471	80.844870580310	85.761084844057
H	115.222290846204	82.126687875794	85.117672889902
H	118.772308037857	74.215600145608	83.007679145814
H	118.637646103478	74.940151480838	76.643966347294
H	113.740999691606	79.032753013142	77.192106173871
H	118.862489076794	75.067060170319	85.169856649031
H	117.747135060029	75.751055379858	86.378220426602
H	118.484167411246	76.801676505998	85.152395241075
H	119.888964219760	71.829858762951	81.262989022761
H	120.477936811889	73.186154624736	82.246792626542
H	121.324054883765	72.718904446290	80.753440230649
H	118.209046012443	75.948651916693	74.481405617110
H	116.671634902403	76.316763998939	73.661428159143
H	116.868814504965	74.794110187088	74.552060902932
H	111.354381127977	80.709794344073	79.943746678386
H	111.719739068524	79.995876600633	78.349101407381
H	112.614621454619	81.412388789394	78.915566245589
H	116.111018009299	77.444551054602	85.139962438321
H	116.480077709801	74.544091364981	84.611467978649
H	120.771595575442	81.812642123795	79.842797571149
H	113.851126814752	75.363340710643	84.719223547387

C4

79

C	105.756642623608	93.136686466482	89.002104118072
C	105.032738394562	87.310355142269	82.030119474129
C	110.446090520111	92.969861586980	80.264883228285
C	110.401280922850	94.546143863956	88.131550415046
N	107.359332207012	90.991751401528	86.970673001271
C	104.212991453281	86.240777483489	81.962530713726
C	109.606881866205	94.243061082010	80.093878014860
C	109.281743715962	93.724176512802	88.891744546697
N	106.766957307214	89.547705158175	84.418353144030
O	110.971762136773	95.509336768823	88.625821705373
C	109.911230735288	93.114080662289	90.133912525385
N	109.536924925885	92.323363714453	85.501174497723
C	111.563728319475	91.551713769060	90.859285247655
C	108.778379539792	92.718743317844	87.871196469059
C	105.674259560600	89.261468011718	86.596074857594
C	107.269281507216	89.481605234096	82.031521917863
C	110.544138467079	92.860255652307	83.359909327503
C	106.247730464842	89.402088003306	89.716996382604
C	104.141147253256	86.983923123581	85.125528468139
C	108.462296326216	90.649591546940	79.343297182146
C	112.191018031946	94.983664149900	85.111239539705
C	107.685051077330	91.875805164710	87.965501082403
C	105.882085353585	88.921649060701	85.248908246212

C	108.297164926348	90.444514166955	81.969622995857
C	110.451918656164	93.041976496548	84.741507213935
O	109.635953566032	93.439046017466	91.275306792207
C	106.639896292837	91.872707822843	89.060441342990
C	105.125554433692	87.922855207602	84.520054684234
C	108.836744127672	91.010060217375	80.746068411250
C	111.124857565004	94.043287881906	85.556641848910
O	110.842645882224	92.205978067349	89.785684705059
C	105.879753599353	90.556581002133	88.780422262528
C	105.518379508412	88.022477334689	83.191695471308
C	109.753106780020	91.973909732209	81.134425962137
C	110.532129084722	93.928641180596	86.816539988871
C	106.297174140529	90.243539336022	87.357489276402
C	106.568122750294	89.047964975976	83.156549806323
C	109.774570107003	91.976484682948	82.582325204373
C	109.573928862868	92.871412046238	86.720306715436
N	108.889950149805	91.032649148495	83.059399982090
Mg	108.384092173632	90.659226237799	85.089365428323
H	106.358655215085	94.028791951921	89.220222850720
H	104.969699712689	93.068039326028	89.763427951732
H	105.383845532244	87.705557687429	81.074351088387
H	110.668959413307	92.529302984002	79.279978655535
H	111.415316835878	93.238003663319	80.708505153296
H	103.795077896888	85.756505809101	82.842575854260
H	103.964530458460	85.802159477958	80.995506866143
H	110.099175500997	94.983073142549	79.443392696715
H	109.430378341621	94.720629353203	81.066932186372
H	108.626659946064	93.999202313167	79.660869866513
H	108.514742051964	94.432567694612	89.232313603822
H	110.980423296155	91.573328496989	91.782588043550
H	112.521226802968	92.070487630588	91.007153390444
H	111.729783390352	90.523099593978	90.526094579668
H	104.881927772093	88.720070923448	87.107964600840
H	106.962469310172	89.060542851205	81.075745822341
H	111.226558175924	93.519092843710	82.820455003489
H	105.635294022755	88.516087604263	89.500709785512
H	106.072792091491	89.687215146024	90.763204475995
H	107.298131914995	89.117906817468	89.589374758312
H	103.113386550943	87.195901108660	84.796721472851
H	104.158886473542	87.035766667172	86.219584450650
H	104.359975999764	85.942198305005	84.848528165723
H	107.780686213334	91.388985182759	78.890383189169
H	107.966622806713	89.670859028342	79.292482820703
H	109.353423680766	90.599762740812	78.697994213641
H	112.081500863974	95.254099634518	84.049995507823
H	113.187599341067	94.532626830386	85.240716031206
H	112.187480110778	95.900598009615	85.714638033286
H	107.103244304700	91.855455313295	90.059414938788
H	104.798916663091	90.722326557531	88.832411064412
O	110.179471427260	88.754603444615	88.305162201042
H	110.768474447020	87.978263993818	88.473901474486
H	109.807934670404	88.996012413517	89.179517159771
O	109.622026674785	89.162081946888	85.796719297116
H	110.536245518252	89.353201916026	85.517640553437
H	109.707781830184	88.998071519603	86.796301302187
H	105.267754421447	93.327668960521	88.010035157977

C5
107

C	93.664723004181	77.445708584619	68.771129618652
H	94.562035987394	77.354401301562	69.399057523949
H	92.891512377411	77.931916981211	69.373923133766
C	93.157705556926	76.129538252837	68.277199472904
N	93.968174550165	75.232250820491	67.610348092242
H	94.972185671315	75.328426649480	67.449604981416
C	93.220168731344	74.188776631993	67.188956243034
H	93.614851340503	73.334044632081	66.649510662244
N	91.954712376309	74.354834347609	67.551962999850
C	91.901549025011	75.558721879307	68.231142399484
H	90.971307800873	75.932793395525	68.646965012988
C	89.577843073264	74.740770148144	57.599058325545
H	89.592934051522	75.671277543082	58.182789990532
H	88.629316704016	74.735994275060	57.047422528235
C	89.631984449127	73.535708181135	58.540771258101
H	90.650248870440	73.357665205794	58.913967706524
H	89.015150450786	73.738859104191	59.430092507748
C	89.102866964231	72.272606839146	57.860436434394
H	88.045209964310	72.430171010970	57.608007811137
H	89.607464984116	72.115932681628	56.894893984114
N	89.248091746365	71.102402849403	58.713101789145
H	90.079441774166	71.077313278024	59.322758690262
C	88.318856034717	70.168627705487	58.952759462008
N	87.162646233867	70.156353705156	58.260569968721
H	86.948444713131	70.943798081605	57.660963925240
H	86.356717004702	69.603540703242	58.583830063871
N	88.582452786861	69.245501275475	59.885345018891
H	89.585216180518	69.098572612825	60.147217265137
H	87.975859414160	68.443567402051	60.045411950584
C	87.964079136543	71.893444352611	62.638862656350
C	87.349391815779	74.565535165603	71.611644097905
C	93.849008017419	69.882594042857	71.262708889917
C	92.167317356909	69.765214270588	63.266447488414
N	89.516538207635	73.007545546652	65.580740637760
C	86.252120525291	75.209781548685	72.060698429175
C	95.059199040603	70.747093412815	71.644657480786
C	91.103670583048	70.858388002632	62.831803951508
N	89.020915438792	73.452500581666	68.500625355786
O	92.544645058672	68.880608105123	62.485471806589
C	91.637959909119	71.701234374168	61.696430243479
N	91.755634736534	71.289355091153	66.418020840387
C	93.251034770063	73.282032295262	61.042507340115
C	90.793225364602	71.599907539569	64.113139254406
C	87.700088590760	74.290624683259	66.607827676342
C	89.840318904503	72.916415090574	70.749395148831
C	93.038587283362	70.249859067257	68.227717997360
C	88.230497416304	75.496236356766	63.629184855336
C	85.722357711863	75.168206472681	68.864504513172
C	91.707795797550	71.556655331717	72.903702132430
C	94.277469807816	68.552238752075	65.842367572482
C	89.795470086505	72.519096520079	64.331677116742
C	87.930928471275	74.091126783456	67.985622459624
C	90.928894785888	72.068731712445	70.441005874925
C	92.715193881079	70.376605891752	66.864472804438
O	91.366797310271	71.537620311798	60.512937899053
C	88.808051346349	73.001262481488	63.290565559921

C	87.070936869971	74.560378736399	69.056451455718
C	91.793160466095	71.418971841735	71.422489121533
C	93.212915190441	69.583682615939	65.766846293182
O	92.507155359218	72.638468314428	62.104218609924
C	87.998411901644	74.054271444572	64.086718702001
C	87.721277558320	74.257677780856	70.247448709026
C	92.708807256444	70.678442679984	70.708729720111
C	92.478974708354	70.015182828725	64.645649984027
C	88.425758950983	73.806645420365	65.523862596245
C	88.936169108351	73.520842126745	69.871125709879
C	92.386923615427	70.871457222286	69.299022220163
C	91.629929278429	71.065056318474	65.109784325771
N	91.307691682037	71.722773521412	69.177156600485
Mg	90.611030405030	72.677149538220	67.442167096746
H	88.578485974299	71.241925153324	62.005903055177
H	87.243642329497	72.378400284824	61.975840648782
H	88.072050278487	74.243247318189	72.365144206114
H	94.170987155880	69.129543871157	70.530091883566
H	93.514011913525	69.326720519731	72.152791939662
H	85.467196529719	75.578843312759	71.402181497050
H	86.116085226478	75.382828933932	73.128376413692
H	95.861290282790	70.130622189904	72.073120188383
H	94.780823044908	71.502011574663	72.392090334738
H	95.459705028589	71.261407149219	70.758252002676
H	90.247477650738	70.299041214313	62.432483440350
H	92.587750315367	73.924537953554	60.452643657779
H	93.703708904006	72.521181042842	60.393433517203
H	94.023551555616	73.860840341938	61.554418773708
H	86.817296830883	74.878349977361	66.349479328851
H	89.664212304432	73.076938639480	71.814108219647
H	93.841950305297	69.555489702340	68.479539696829
H	87.629857858284	76.204496152911	64.216640132537
H	87.957962529385	75.616125236465	62.571073358343
H	89.285569479247	75.775339095435	63.749258910312
H	85.410897217505	75.104417930935	67.814384123980
H	85.683926135675	76.228441385412	69.156793131547
H	84.961079810292	74.651009206721	69.469370926736
H	92.301248450828	70.784464471374	73.410463658995
H	90.672038446879	71.478205819829	73.265423413381
H	92.102197253313	72.529414596246	73.232532609775
H	94.827456481944	68.494774076761	64.894195423001
H	93.858084928110	67.556638327094	66.061191372909
H	94.996765766819	68.782389214693	66.638206175366
H	89.341000000000	73.467000000000	62.436000000000
H	86.935000000000	73.827000000000	64.029000000000
O	91.264107715861	68.685785072229	60.120002850295
H	91.172342632366	67.719172715537	59.998245874916
H	91.744587932533	68.793309095972	60.994539062445
H	93.924641603743	78.137043396562	67.932986809437
H	90.439381521685	74.778351969544	56.887093136813
H	87.372890734290	71.218220336778	63.301388860486

C6

85

C	107.143995063652	60.720112847273	64.958542399277
H	107.745781255096	60.863921916631	65.862975172716
H	107.308325167790	61.583058826800	64.303823259606

C	105.701274850629	60.588193219663	65.294442355566
N	104.740654688500	60.326493117567	64.328026033183
H	104.911245497922	60.060825829122	63.349524123210
C	103.536767123945	60.232252514454	64.936447464451
H	102.611988306310	59.997600123119	64.418769249022
N	103.660572438269	60.451605855827	66.237943895588
C	105.002749624446	60.675931466224	66.475881298176
H	105.384678278917	60.870453849887	67.472681098861
C	97.539667998666	61.092761914117	64.740941401694
C	103.561055074686	65.396234549929	70.026833470045
C	104.178281827148	57.616628808090	71.901224925327
C	99.583698595029	56.486191617809	65.221253026195
N	100.633099737692	61.152499931873	65.934438204330
C	103.965721099741	66.672630049993	69.889327826376
C	103.063561690472	57.249879195490	72.895165852427
C	99.353353851735	57.837416618761	64.402922564872
N	102.249984079938	62.587313702329	68.005401797398
O	99.129457397233	55.409515538908	64.837410695437
C	100.084087698535	57.669927053899	63.076449501801
N	101.287553784762	58.683412068110	67.409938758698
C	102.204542832028	56.989685555622	62.166773120357
C	99.944173740312	58.930292528971	65.287741740225
C	101.102693083583	63.559639922613	66.062031066515
C	103.544763472000	62.285755352640	70.066919724386
C	102.407506705715	57.613030688479	69.302857467558
C	100.590025162971	62.689738675670	62.985713319395
C	102.156125865336	66.253022024155	67.275907825816
C	105.094457698128	60.684052963690	72.282574076210
C	101.179405257841	54.956728470657	67.985739899639
C	99.900265120666	60.306223333640	65.141522524193
C	101.881192066119	63.652138068504	67.237291624823
C	103.503356903389	60.880951115351	70.204186278032
C	101.612808450162	57.542375359710	68.142526444467
O	99.576377362625	57.788903089825	61.968322797351
C	98.993956096055	61.102332850059	64.216612267050
C	102.363656372660	64.890076468482	67.827221471595
C	104.173000673976	60.118340174880	71.251235337839
C	101.052229276749	56.365205014688	67.513799429242
O	101.366182733605	57.323189549769	63.297804014760
C	99.687693409827	62.486872382476	64.210960065106
C	103.004790392959	64.537053487465	69.004075048089
C	103.821536471379	58.798753876498	71.055310708940
C	100.392087647803	56.845126130222	66.367074203499
C	100.517815764961	62.432549356838	65.493964729519
C	102.944552476357	63.076113169580	69.085598770007
C	102.962917990759	58.765984791847	69.874403515267
C	100.578658425910	58.261397030368	66.361954721939
N	102.799963777378	60.045840489132	69.386280792008
Mg	102.001905312525	60.605271232055	67.546155267534
H	97.157906175168	60.063586631361	64.759944931744
H	96.882668394026	61.667000759352	64.072911844202
H	103.641197104300	64.947368193724	71.022259831565
H	105.106107137946	57.821877211364	72.456954164609
H	104.393612018153	56.747007355794	71.258101588397
H	103.955789623262	67.206375091556	68.941528323199
H	104.342930288852	67.211680922389	70.753118713142
H	102.857670883239	58.089029172012	73.574099590340
H	103.347101512823	56.378561107979	73.503090489319

```
H   102.127038756697    57.013128047490    72.370603182131
H    98.286400516979    57.957613399272    64.168001901856
H   102.633830944542    57.908084051586    61.744427916515
H   101.638544576703    56.444432316676    61.403810913858
H   103.005657876609    56.365780181647    62.573078735212
H   100.902524689924    64.502575882586    65.549779113814
H   104.118887298771    62.816805695351    70.827617930714
H   102.628683597333    56.668277086755    69.802948712285
H    99.993741449417    62.717740899717    62.059744248524
H   101.321022607869    61.870884395145    62.901854487129
H   101.149137374396    63.632148466922    63.062928392280
H   101.856060985902    66.953280012776    68.069779519474
H   101.382545588486    66.259912338417    66.501049431441
H   103.078756771725    66.649354579781    66.821536460738
H   105.490260963587    59.898712466390    72.936966265862
H   104.609803309184    61.436030449193    72.919911820174
H   105.954690620179    61.187002577095    71.812846863046
H   102.208590385892    54.586709290762    67.853530423349
H   100.512338904901    54.296499118781    67.417860176612
H   100.935764412245    54.864409646029    69.054967042778
H    98.970875935791    60.668036288856    63.203094236740
H    98.958481553036    63.311369184712    64.252337097204
H   107.486993266825    59.795057427159    64.450582389277
H    97.402577143414    61.503222004996    65.773127296058
```

C7

```
82
C   101.812000000000    74.147000000000    67.884000000000
H   101.678000000000    74.825000000000    67.025000000000
H   102.382000000000    74.635000000000    68.685000000000
O   102.459000000000    72.928000000000    67.508000000000
H   103.401000000000    73.118000000000    67.316000000000
C   104.465000000000    72.010000000000    70.146000000000
C    96.892000000000    66.752000000000    66.698000000000
C   102.306000000000    66.972000000000    60.799000000000
C   107.093000000000    70.352000000000    66.415000000000
N   102.607000000000    69.741000000000    68.062000000000
C    95.974000000000    66.214000000000    67.527000000000
C   103.085000000000    65.650000000000    60.887000000000
C   106.363000000000    70.648000000000    67.781000000000
N   100.103000000000    68.580000000000    66.782000000000
O   108.288000000000    70.553000000000    66.225000000000
C   107.059000000000    69.967000000000    68.951000000000
N   103.884000000000    69.205000000000    65.465000000000
C   107.942000000000    67.920000000000    69.723000000000
C   104.917000000000    70.184000000000    67.564000000000
C   100.389000000000    69.448000000000    69.065000000000
C    99.267000000000    67.380000000000    64.815000000000
C   103.723000000000    68.445000000000    63.150000000000
C   102.129000000000    69.687000000000    71.612000000000
C    97.480000000000    68.365000000000    69.445000000000
C    99.330000000000    66.348000000000    61.840000000000
C   106.803000000000    69.323000000000    63.066000000000
C   103.846000000000    70.194000000000    68.449000000000
C    99.647000000000    68.819000000000    68.049000000000
C   100.362000000000    67.541000000000    63.939000000000
C   104.456000000000    68.988000000000    64.214000000000
```

O	107.343000000000	70.497000000000	70.006000000000
C	103.855000000000	70.621000000000	69.913000000000
C	98.326000000000	68.242000000000	68.223000000000
C	100.429000000000	67.045000000000	62.573000000000
C	105.853000000000	69.381000000000	64.216000000000
O	107.312000000000	68.670000000000	68.665000000000
C	102.365000000000	70.466000000000	70.317000000000
C	98.027000000000	67.583000000000	67.038000000000
C	101.694000000000	67.348000000000	62.109000000000
C	106.088000000000	69.831000000000	65.516000000000
C	101.727000000000	69.843000000000	69.087000000000
C	99.152000000000	67.833000000000	66.132000000000
C	102.384000000000	68.028000000000	63.189000000000
C	104.855000000000	69.718000000000	66.229000000000
N	101.541000000000	68.154000000000	64.274000000000
Mg	101.920000000000	69.221000000000	66.047000000000
H	103.956000000000	72.750000000000	69.515000000000
H	105.531000000000	72.022000000000	69.895000000000
H	96.798000000000	66.526000000000	65.632000000000
H	102.972000000000	67.778000000000	60.451000000000
H	101.517000000000	66.896000000000	60.037000000000
H	96.004000000000	66.349000000000	68.606000000000
H	95.186000000000	65.566000000000	67.143000000000
H	103.912000000000	65.731000000000	61.607000000000
H	103.505000000000	65.352000000000	59.914000000000
H	102.428000000000	64.839000000000	61.235000000000
H	106.430000000000	71.731000000000	67.963000000000
H	107.197000000000	67.643000000000	70.481000000000
H	108.729000000000	68.517000000000	70.200000000000
H	108.354000000000	67.029000000000	69.242000000000
H	99.858000000000	69.575000000000	70.007000000000
H	98.417000000000	66.827000000000	64.412000000000
H	104.255000000000	68.305000000000	62.207000000000
H	102.503000000000	70.244000000000	72.478000000000
H	102.638000000000	68.716000000000	71.586000000000
H	101.061000000000	69.491000000000	71.773000000000
H	96.419000000000	68.479000000000	69.172000000000
H	97.766000000000	69.237000000000	70.048000000000
H	97.549000000000	67.477000000000	70.092000000000
H	99.308000000000	65.267000000000	62.054000000000
H	99.441000000000	66.455000000000	60.751000000000
H	98.340000000000	66.735000000000	62.121000000000
H	106.979000000000	68.293000000000	62.723000000000
H	107.770000000000	69.756000000000	63.346000000000
H	106.411000000000	69.875000000000	62.198000000000
H	104.454000000000	69.872000000000	70.457000000000
H	101.925000000000	71.473000000000	70.428000000000
O	101.535000000000	71.215000000000	65.753000000000
H	101.875000000000	71.854000000000	66.449000000000
H	101.197000000000	71.796000000000	65.048000000000
H	100.819857000000	73.894143000000	68.292571000000
H	104.396429000000	72.428571000000	71.176000000000

C8

88

C	96.212002418105	79.573444006904	62.726824265818
H	96.113178490932	79.171658970183	63.743477333323

H	96.766303091423	78.849494049062	62.110145787337
C	96.923622617470	80.880380448324	62.780137489744
N	97.290614739638	81.588693347005	61.647702541293
H	97.065456049817	81.382873268754	60.658365867204
C	97.945567841841	82.702960902311	62.041793076729
H	98.324659252607	83.447222343743	61.350921990609
N	98.026124902752	82.762254499137	63.368086677330
C	97.383898801690	81.634243241325	63.838002395982
H	97.271210443107	81.439656121796	64.897820679919
C	101.457741777628	87.001491646284	60.252839364510
C	102.481829676886	81.821146373134	67.911233482247
C	95.409052594277	85.641358384256	68.572898139744
C	96.599665339080	87.551910979383	60.771037925856
N	100.189131778642	84.835239581554	62.521470963150
C	103.416114662022	80.860629813172	68.051847495454
C	95.412639487147	87.070256740687	69.128121627133
C	97.848950374349	86.812720270355	60.149636872009
N	100.692404832293	83.611787846044	65.200616602749
O	95.830012543002	88.208013142264	60.078970844546
C	97.366373793643	85.751640470934	59.178522064463
N	97.709433828616	85.906378845323	63.698622504049
C	98.042210651224	84.184333704278	57.538216839408
C	98.537441251113	86.130388696877	61.331071361243
C	102.138010615343	83.520286189886	63.213641863934
C	99.843533759284	83.441915662462	67.496023259050
C	96.276009274619	86.189416300476	65.649060211453
C	101.771929325474	83.195070012937	60.067890959849
C	104.010455439980	81.886093717058	65.095200863862
C	97.972266600116	84.095925411925	69.906078921449
C	94.699804161751	88.181897223101	63.698597970493
C	99.769933424383	85.502377587687	61.400593033385
C	101.820812307146	83.202805736057	64.551125854634
C	98.651588529355	84.187988477859	67.383586141046
C	96.603090241331	86.483103241206	64.317251574698
O	96.252952141698	85.244467076146	59.157786481217
C	100.879783350124	85.574705510895	60.367653869902
C	102.670513608496	82.435287951707	65.440986309178
C	97.735790761474	84.473675979985	68.482140448089
C	95.888083006154	87.337244275466	63.390438892802
O	98.344135658434	85.365491966515	58.321513077966
C	101.867697884314	84.499330629660	60.871904662512
C	102.030759263795	82.418326131139	66.672241283969
C	96.685575608536	85.184271915774	67.936135550068
C	96.580943772152	87.208523444678	62.177666067488
C	101.408472137819	84.275792431574	62.307160157270
C	100.786135956169	83.172696645970	66.501279624555
C	97.011481687356	85.384861078583	66.530345119806
C	97.677379769324	86.328955150925	62.435756013581
N	98.189134918244	84.738346315387	66.222162419868
Mg	99.007819041257	84.434084875537	64.326663216291
H	100.781614209792	87.624998767520	59.657823824739
H	102.429245933813	86.973239430838	59.743207090113
H	101.976491557680	82.171686483598	68.817377289024
H	95.145300351088	84.952556251882	69.382403617216
H	94.606526950065	85.550753358355	67.823134597204
H	103.941138668704	80.410678259883	67.211806009940
H	103.647369076652	80.447582968084	69.031655158743
H	95.742226406897	87.794754933054	68.369600284953

H	96.081843167036	87.152600721024	69.993209628167
H	94.405374395866	87.357407869398	69.462529120534
H	98.482496355699	87.546358211484	59.629438435790
H	98.943727578396	83.984958017158	56.950542937644
H	97.185810385790	84.373120486768	56.877572044758
H	97.795348417691	83.335542741532	58.187466448511
H	103.084759373803	83.124524492809	62.841696280288
H	100.060107234242	83.048292351553	68.490596453039
H	95.368432944971	86.647426199911	66.046086992700
H	102.081397546475	83.349734877806	59.023181749080
H	100.736513176952	82.821522219572	60.062375151643
H	102.402019608802	82.409215796096	60.506349506459
H	104.727011765952	82.053474778908	65.913109815616
H	104.416334208157	82.350607486416	64.189044790393
H	103.986168030329	80.801081462781	64.910985616167
H	98.877577691644	84.586204651094	70.297488950333
H	98.132226764660	83.014836885961	70.035926576725
H	97.134321192773	84.396348761021	70.542568724118
H	93.915418144755	87.607812635370	64.212541539343
H	94.269134518186	88.590777532283	62.778737897808
H	94.971482050503	89.023379031524	64.356954513510
H	100.490421039635	85.298870616866	59.379877256775
H	102.906996527486	84.865535108672	60.846843030177
O	98.801903198594	89.723680458424	61.338288438225
H	98.838886868992	89.379982738160	62.245688036978
H	99.404622637301	90.497010655249	61.335295438262
H	95.191117856033	79.697669909424	62.308198212604
H	101.598620984311	87.518738624495	61.229843402774

C9

91

C	112.231601448480	79.264739847073	64.394074052524
H	112.998091165317	79.124943672995	63.618893753726
H	112.408587807293	78.524307888592	65.190048746320
C	110.846874582298	79.126000870546	63.851681136882
N	110.494257402590	78.390640522974	62.731256027769
H	111.111207268865	77.951245264970	62.038809195305
C	109.138658117228	78.403513875809	62.627255125895
H	108.594841417204	77.914032142007	61.826052637772
N	108.598829921982	79.100337501000	63.618869199187
C	109.651331769031	79.552164155086	64.383382416413
H	109.480113587685	80.149756683517	65.273921559346
C	101.737283822947	74.257645906570	67.813923610687
H	101.511632305577	75.041848014882	67.071179085803
H	102.388316818007	74.676979904787	68.595757792804
O	102.343825338913	73.116914119191	67.218749626018
H	103.287457544627	73.336817798885	67.055446678858
C	108.369448199811	77.964339331199	69.043654870921
C	108.033150601967	84.796551539228	62.394207375087
C	103.992476843215	78.732766176255	59.189442994528
C	105.202436290460	75.190134242653	66.493152876580
N	106.931073420848	79.687604616075	66.369614049484
C	108.679861123884	85.971123892270	62.528504983203
C	102.503688160903	79.115609014666	59.148991109762
C	105.704155383536	76.233180320606	67.580140568793
N	107.040726337526	81.759220924879	64.221116777643
O	104.932707953376	74.019545492861	66.755393406048

C	104.527971157878	76.439222349236	68.533912502526
N	105.604514673066	77.971158625926	64.345165611849
C	102.459274623723	77.605287977364	68.766141237097
C	106.097009183363	77.464122972263	66.776270755269
C	107.855965380460	81.954399050223	66.527780076374
C	106.400721834216	82.241558466158	61.899634639240
C	104.784742167117	77.646136820914	62.068486364568
C	106.933948883159	81.305805763645	69.467866982417
C	108.913972930841	84.729349736201	65.521560328763
C	105.128380381130	81.679811619242	59.055328675379
C	104.215595832075	74.668480692938	63.156902943291
C	106.723465535053	78.622319213141	67.211379389623
C	107.677582172963	82.443699191458	65.219135092223
C	105.759307142588	81.039866395034	61.531517833552
C	105.043046964728	77.161445089999	63.360722253067
O	104.339891129355	75.794138609783	69.538314639289
C	107.235496024175	78.905254300270	68.614448409079
C	108.131810121651	83.732973798320	64.732464746417
C	105.177159332786	80.756540418137	60.230346317233
C	104.756311131358	75.842476165967	63.897855063187
O	103.678298773397	77.378587802944	68.033455113514
C	107.690667028655	80.381916589961	68.520187413359
C	107.737797628665	83.805480410421	63.400257290329
C	104.728155098268	79.450946185885	60.276899304862
C	105.135713624532	75.915686741876	65.243072820050
C	107.484893094310	80.718223116028	67.056198686168
C	107.024153135208	82.562140743926	63.109625030267
C	105.045206950861	78.947298757657	61.602952908355
C	105.648766598862	77.231360215439	65.455865407331
N	105.665438326910	79.937744107204	62.335190885141
Mg	106.614478585648	79.738369081811	64.195151986497
H	109.263897096333	78.163039928658	68.437426495030
H	108.094519477422	76.908660820541	68.928085872831
H	107.733681633127	84.501751987896	61.385344124117
H	104.086874507585	77.643530265600	59.313049946430
H	104.459769315797	78.968925560375	58.217940476420
H	109.018989209782	86.350191834923	63.488841409431
H	108.910378634755	86.579413634176	61.656408739166
H	101.996057136217	78.756073886322	60.054033353039
H	101.999988997483	78.690374613711	58.269011541515
H	102.379087797426	80.206825950831	59.109806610842
H	106.502757080476	75.770123734251	68.173330821101
H	101.652397113216	77.656093222481	68.026920646564
H	102.543595859585	78.564481544570	69.294010540016
H	102.281823713996	76.797929875915	69.486061049804
H	108.335330709960	82.630203730652	67.237732213157
H	106.430174695979	83.010320200399	61.126438164718
H	104.306545064049	76.960368557857	61.366025579306
H	107.199581036716	82.359681054657	69.319211562234
H	107.139903678574	81.039090411653	70.513903858036
H	105.854776002079	81.211064278766	69.314788057929
H	109.946732196230	84.820467933898	65.146287088284
H	108.974309713535	84.444891977532	66.579408170652
H	108.473992790514	85.736500382439	65.472336001677
H	105.758664425791	81.307755995877	58.229395277121
H	105.483702846132	82.686851531522	59.302145903448
H	104.107896338866	81.780850013641	58.655425361860
H	103.564221395673	74.066090751075	63.805643265896

H	105.033972402959	74.004978976502	62.830657654182
H	103.656770062075	74.974102808786	62.261821251141
H	106.388851306823	78.809981470388	69.313526571042
H	108.768165185229	80.463389362412	68.735808775228
H	112.380159215574	80.277277827089	64.825761495171
H	100.784604345917	73.948548286209	68.293694766532
H	108.695082225093	78.088874807682	70.096877951636

C10

85

C	111.615243175576	83.717566247831	68.181911071621
H	111.451641991399	84.436354573578	67.369275283954
H	111.701146574917	82.721485486337	67.729499622508
C	110.502513983314	83.786948425552	69.164529658056
N	110.179743395754	82.738458497861	70.004237697251
H	110.595654588335	81.800058503892	69.942617351387
C	109.219403582849	83.160992889117	70.859450442379
H	108.756759508627	82.544843661817	71.623176965274
N	108.892751826073	84.420180649926	70.601348961932
C	109.686370760259	84.825468247316	69.552200976809
H	109.619407926865	85.832092489368	69.151389597410
C	105.557872703878	85.773586606752	66.559200732767
C	106.103381266142	81.208136205444	74.929164637788
C	110.955570288194	87.524271418606	75.681510169247
C	107.634237448489	89.871530416990	68.548515250522
N	105.959011361792	85.586184974187	69.983078641957
C	105.517527445828	80.003353028577	75.079641893105
C	110.316483427499	88.338322837972	76.817639836145
C	106.696272280246	88.743282086212	67.906516426828
N	106.399506415630	83.992100137092	72.501994589038
O	107.831847229185	90.953776043531	68.004925163651
C	105.330496593975	89.263901741858	67.530233280090
N	107.995772512512	87.425683332723	71.080434239714
C	103.228046091317	89.890274965045	68.381166094752
C	106.681064754371	87.623001227952	68.933017258444
C	104.822418335161	83.536094495298	70.668781503217
C	107.701746613823	83.844459395058	74.579536430574
C	109.547010939560	88.009657153604	72.881544787205
C	102.707046237174	85.107012616953	68.878086693595
C	104.384264567020	80.853481544183	72.260936399050
C	109.767839587178	84.778846953234	76.758719797216
C	109.891786596982	90.679318470555	71.129024407975
C	105.903354481002	86.481688865653	68.950314271941
C	105.475222358018	83.204006325793	71.876542984137
C	108.472442737168	85.019874931608	74.482228292146
C	108.902441196822	88.308518693788	71.663690349427
O	104.918090725747	89.416612687218	66.392273921411
C	104.877063253695	86.073163107012	67.902658175375
C	105.261686072041	81.990180034226	72.640249084011
C	109.445845411836	85.462619527834	75.471104450316
C	109.031515241018	89.498912829054	70.851486514699
O	104.590898485967	89.518301583910	68.635601374244
C	104.185682576950	84.854219375466	68.567582040917
C	106.042519330089	82.090428443435	73.784058893926
C	109.972988182950	86.639402453834	74.988742651734
C	108.158788212419	89.297149161751	69.767362200861
C	105.024629744073	84.620824306231	69.821217501735

C	106.763237853211	83.358373512065	73.669946773731
C	109.320567113143	86.900806836959	73.708104180245
C	107.571640400867	88.012891181296	69.959333584979
N	108.416995624138	85.894861776216	73.435841930389
Mg	107.409602335674	85.552079254966	71.632939578429
H	106.160669333797	84.861022573489	66.661738806451
H	106.224244210842	86.596178305021	66.267125184016
H	106.635815048407	81.605984650025	75.796266804270
H	111.414942952561	88.198844939554	74.946078093523
H	111.781210817733	86.916068323874	76.080653757027
H	104.946874281066	79.507873007634	74.298414564016
H	105.563220900721	79.495483252699	76.039545936428
H	109.420708595792	88.868833560599	76.467357716030
H	111.018714684249	89.078231037358	77.226450781338
H	110.011555048545	87.683233286385	77.644570025339
H	107.171306984753	88.448595620409	66.957181598239
H	102.704995586577	89.085532909422	67.850411653266
H	103.187852203458	90.801188426205	67.770511035641
H	102.781522075091	90.056234129058	69.366304868721
H	104.063011535767	82.831613025322	70.331788432353
H	107.864270848706	83.248150386287	75.477739613067
H	110.285135475141	88.732839001217	73.233540190324
H	102.586897417226	86.055411038610	69.420684592162
H	102.286644707026	84.308366852235	69.504751978703
H	102.125325753123	85.158997659778	67.948386498064
H	104.958537039434	79.922970559762	72.131220038392
H	103.861456508810	81.048028448139	71.320214292270
H	103.628656314341	80.658221426026	73.036040326193
H	110.696370855313	85.168589561703	77.197680098770
H	109.886626203934	83.692997181348	76.631648911854
H	108.971423221429	84.918420855446	77.504116537561
H	110.272873902886	91.110403920211	70.193415364173
H	110.744249056025	90.428183178338	71.771118844313
H	109.340495532760	91.476540998321	71.651445972294
H	104.151813900151	86.891345415200	67.778887542657
H	104.258726717105	83.967614038061	67.918104740420
H	112.594926852982	83.986136670314	68.632756357964
H	104.892823758290	85.588013289713	65.685654169236

C11

85

C	105.597287991285	86.918990609775	60.499319014152
H	104.596190950549	86.917473342008	60.943107274287
H	106.040369658541	85.925893330988	60.663671234191
C	106.508435683942	87.943797121369	61.142827392460
O	107.577930942663	88.260368956548	60.577990370471
N	106.125967398083	88.447261851872	62.318126495001
H	106.737669039535	89.099547197024	62.810240560975
H	105.310083856831	88.080653566474	62.812168629363
C	112.912016724884	89.037603990758	56.719402834903
C	110.200040596092	88.931077857412	66.070296308015
C	105.338077991612	94.006308652806	62.299788106227
C	108.620551318019	90.881914946497	55.486768830767
N	110.549128109703	88.946756842443	59.474716587810
C	110.531966682556	88.004718564601	66.989814952860
C	106.047339163167	95.355661494512	62.513922875699
C	109.794615158684	89.848593288003	55.799170919021

N	109.935999072977	89.290682381082	62.390816277866
O	108.370533965806	91.287234321655	54.345676962663
C	109.609993063962	88.556108324238	55.035508667165
N	108.282987624334	90.801751589057	59.002265544191
C	109.987156458204	87.489738863011	52.946882135620
C	109.784678437088	89.680816742584	57.309878492639
C	111.709409638837	87.931377884878	61.382888010637
C	108.501022915276	90.471123697303	63.999364479759
C	106.704093026303	92.437053907297	59.917292247096
C	113.091225760921	86.394549807741	59.034356298979
C	112.272632055756	87.153693121010	64.406238175812
C	106.373311765536	92.554780303917	64.956350559928
C	106.318321865173	93.144402352701	56.792489244671
C	110.696137631268	89.018926903488	58.111371294619
C	110.975890139018	88.408666076225	62.485734861662
C	107.732186977239	91.266835551756	63.121279311356
C	107.332301294314	91.808911609623	58.821966208520
O	109.041685087633	87.568587537350	55.456361642213
C	111.944338186894	88.281197228137	57.648078122104
C	111.234182773110	88.083507830491	63.883996808047
C	106.749430088104	92.256054937404	63.542885885393
C	107.178249315492	92.104271204469	57.417739533316
O	110.169954291310	88.642853287959	53.800826340214
C	112.595490151281	87.836275164154	58.982692007018
C	110.331922533853	88.823732206538	64.629679231999
C	106.275000805451	92.843361591291	62.390223340263
C	108.049100498885	91.215650075974	56.760908782599
C	111.560260272653	88.233385392807	60.028448678838
C	109.525217892364	89.581524063029	63.670779223686
C	106.936774761350	92.176387963313	61.272839591068
C	108.713954415777	90.473618605511	57.783988908611
N	107.820638681149	91.228057868791	61.757113080475
Mg	108.921691713209	89.794289232058	60.669894830713
H	112.547424478273	89.071499012919	55.683902717548
H	113.883485403046	88.520589138908	56.711387913400
H	109.787532811894	89.877204985992	66.430013950530
H	104.559528857025	93.892992306801	63.068597571169
H	104.825480384188	94.000734529107	61.324823575880
H	110.917909256246	87.021476622336	66.721215647916
H	110.402716332154	88.210547080311	68.052435497658
H	105.396209570343	96.196947846107	62.239130837088
H	106.965621218497	95.418718705962	61.914315651815
H	106.323412416092	95.492103225589	63.567649590742
H	110.707671209620	90.331791093699	55.422732270547
H	109.812036131299	86.602918342735	53.563567168734
H	110.897553383205	87.389318305954	52.347848040279
H	109.124897673863	87.666605328120	52.292690349782
H	112.548109545160	87.278075696872	61.624762935274
H	108.288866080041	90.597916422139	65.062407519607
H	105.958287761114	93.198945161865	59.683824659216
H	113.636508549271	86.190513597806	59.965844788535
H	113.788188785977	86.193526894826	58.207145271137
H	112.269633554726	85.670511426269	58.980739562268
H	113.063551537961	86.967868629127	63.672434550023
H	111.848181488451	86.175581957123	64.682613462382
H	112.743096895958	87.558732965627	65.313850974606
H	105.840856773628	91.707663590809	65.414469997206
H	105.724176256595	93.437524340918	65.021105353889

H	107.259563312111	92.745133612049	65.579495906342
H	106.900774632628	93.759313220208	56.095583190412
H	105.844262330299	93.797457374333	57.532810583875
H	105.524269801286	92.681080891433	56.188170222436
H	111.613144745089	87.400975662132	57.078335743598
H	113.477936313770	88.476452294167	59.150711738504
O	109.560329068426	94.930195815086	53.347420370291
H	108.812954711720	95.330338270332	53.837644256609
H	109.137668295588	94.232907729936	52.801741362452
H	105.548358452107	87.098273617295	59.399615265200
H	113.093600037826	90.084645046364	57.043624327252

C12

88

C	110.874191475704	68.427061914790	68.297187407198
H	110.009632418263	67.927086050062	67.835627805046
H	110.506537350906	69.054643536241	69.124720375456
C	111.635983926243	69.241942967747	67.301353943674
N	111.018975112665	70.129061379565	66.443442462243
H	110.012951792232	70.291065489010	66.370297781700
C	111.958880475450	70.723119409333	65.672760121747
H	111.740795585147	71.473620718584	64.920065786883
N	113.164258347922	70.264244208239	65.986331788871
C	112.978208590687	69.348961741470	66.999701714543
H	113.822693058932	68.828236547543	67.441872950393
C	115.025814455289	72.871781918308	70.532273444832
C	118.131951499006	66.496456532865	63.973880770229
C	114.273716603513	72.059587684490	59.705258521065
C	113.695651362981	75.955150000336	66.790377708368
N	115.720243394032	71.621228333173	67.324391961116
C	119.193645644076	65.721011823427	64.267538961429
C	115.238640987690	73.196753190825	59.331437634896
C	114.239798587182	75.116194040381	68.027107593621
N	116.308791428871	69.403447922679	65.377941140987
O	113.339450245874	77.129248788909	66.889389439865
C	115.276563341122	75.937966582392	68.760028996517
N	114.368661390489	72.955275458839	65.052014786582
C	117.462562084203	76.805858049419	68.694628916134
C	114.676504034063	73.787776061456	67.425067565081
C	116.954973103950	69.549734529143	67.746789138451
C	116.327322248034	68.826597459965	62.995379766435
C	113.941933593279	73.055442756523	62.647275828463
C	118.411150582551	71.793937391356	69.408474398199
C	118.331439849672	66.824436708201	67.179878508682
C	115.766506551047	69.200288665449	59.948153627325
C	112.891019190890	75.989064345327	63.370894122415
C	115.416698650002	72.772809643616	68.005615313509
C	116.894405862081	68.894152132184	66.503195430213
C	115.602393571977	69.911640276027	62.463552077239
C	113.889772500118	73.627941906748	63.929830783236
O	115.085616204021	76.549023231945	69.794569114827
C	116.047701928422	72.793380436054	69.391380988402
C	117.628591153636	67.683138525712	66.187080109719
C	115.353097011222	70.127945962125	61.045169592789
C	113.461539602207	74.962775406810	64.282588144805
O	116.459805127402	75.968670818758	68.092287048927
C	116.907098875100	71.501806762332	69.382920549554

C	117.525317015911	67.507755479927	64.814991135924
C	114.684690590619	71.330761364551	60.945284109628
C	113.725101496121	75.069633064440	65.656725516102
C	116.499603850722	70.822164527827	68.086942952515
C	116.671990362204	68.595420101917	64.327756905433
C	114.526187835006	71.828720159324	62.305968952752
C	114.271517842692	73.817211969802	66.068289834156
N	115.084663659401	70.938743740069	63.200486333188
Mg	115.027697230826	71.027068326729	65.312947135765
H	114.458796342130	71.933431203873	70.599579566839
H	114.304318230635	73.683995905198	70.384062723978
H	117.669365995465	66.358809680198	62.993073409568
H	113.259307067330	72.472712460501	59.828671060588
H	114.215577718486	71.355874596143	58.863251007055
H	119.745577274640	65.807813453679	65.202310284652
H	119.552415825658	64.976569231328	63.556518579261
H	114.942519408361	73.663213617234	58.383149200869
H	116.261513754458	72.812647427715	59.209000081453
H	115.265904033091	73.967341335210	60.114171848123
H	113.390966147643	75.009128629824	68.722347925557
H	117.088416471971	77.833026186883	68.801285548557
H	118.320648212442	76.770665195987	68.015398163753
H	117.739869378894	76.428255009270	69.688523264698
H	117.543418696237	69.044358625229	68.514444040933
H	116.696694303950	68.102362884613	62.267503902607
H	113.535874832560	73.648204600086	61.825978026398
H	119.004228865334	70.877110082257	69.291913611510
H	118.689701960239	72.264384093732	70.358473388410
H	118.680480552266	72.482225771232	68.594985549769
H	118.267455883593	65.767416822162	66.882537604275
H	117.891079192998	66.925315233086	68.181201854990
H	119.403170131609	67.072447556387	67.265725895656
H	116.855525473439	69.211003638991	59.776361928642
H	115.277355149706	69.455994915868	58.999087751554
H	115.508454601381	68.155268092878	60.177903034303
H	112.674062685752	75.590103621725	62.373375679845
H	113.580115999246	76.839335071631	63.258683714382
H	111.961113637788	76.397468856193	63.788812404835
H	116.713227530196	73.672273150852	69.444357013974
H	116.661290064781	70.855557069238	70.239855441473
O	112.210408726182	77.492103475710	69.424332730205
H	112.933247224305	77.285525245109	70.043619301448
H	112.654121794288	77.461156701394	68.544723941666
H	111.536522988710	67.641415985472	68.712488074945
H	115.512287775048	73.070129416747	71.512756623173

C13

101

C	123.539516043735	83.991346107025	60.861465912903
H	124.324448950527	84.582327176024	61.352340500307
H	123.068696463805	83.360591053402	61.625756407981
C	124.056189892033	83.140776609506	59.724518477926
C	124.927743149147	83.650070318217	58.750067371571
H	125.357250855329	84.645840353859	58.890202401584
C	125.244164300259	82.922736320553	57.601904734320
H	125.907650759211	83.334014361312	56.838461678858
C	124.704731740178	81.642175972526	57.414282301720

O	124.993648253728	80.978560868099	56.255202503599
H	124.539905715427	80.102575721665	56.281564260944
C	123.892078628689	81.088039492858	58.412494475652
H	123.512825640215	80.071648415821	58.298545244613
C	123.575740804892	81.835352755153	59.544956528254
H	122.920319619897	81.393403149915	60.299650951505
C	119.014569482519	81.534698093669	59.833898275720
H	118.849244216666	81.855509703385	60.866250325472
H	118.050608324771	81.282040358820	59.366869115083
C	119.907444069254	80.348156397084	59.830514847026
N	120.201167370213	79.645118854376	58.675726660466
H	119.827377443926	79.885557585206	57.748935547809
C	121.016713256703	78.612467739821	58.999016491974
H	121.433115517149	77.917803158608	58.277019110300
N	121.256952808539	78.609034075429	60.306578512754
C	120.571324714289	79.684546058850	60.835526153093
H	120.601049092224	79.916011589365	61.894611788942
C	123.986724375293	73.430878758288	57.820885221995
C	118.423214229173	75.474901200445	65.015747275754
C	124.952124591120	80.024462137468	65.731438662514
C	126.425917865814	77.908668110625	58.050263220806
N	122.465307703204	75.736791789824	59.815685863060
C	117.519033482869	74.565693614815	65.424406881697
C	126.115523100338	79.142446566525	66.212910930015
C	125.340689515881	76.843310479153	57.487510225702
N	120.903438696495	76.102155765566	62.343032328998
O	127.402947515074	78.263419387756	57.397523249087
C	124.671362876286	77.538256130044	56.330111556233
N	124.291909199985	77.796745641164	60.871804485300
C	124.574781313514	77.886619216090	54.006537812257
C	124.391047812741	76.606313953312	58.651210090375
C	120.432701706491	74.545870900041	60.505514482655
C	120.930164022473	77.199277887249	64.536019786341
C	124.954499051377	79.249186850243	62.716254698028
C	120.463039131115	74.742773094470	57.310969346152
C	118.101486175168	73.612705551942	62.376927251872
C	122.286862544724	78.705713870743	66.949722803390
C	127.241523570147	80.127672793479	60.663496621572
C	123.331758300678	75.719242869613	58.750992965688
C	120.172859041671	75.097599318223	61.775087732226
C	122.112797519658	77.961755397760	64.431456259025
C	125.139792979276	78.760428389667	61.411595933593
O	123.805561315681	78.403145545705	56.445499658900
C	122.984375242825	74.596121498124	57.790478012394
C	119.100361571381	74.681454268307	62.664926233317
C	122.752192554292	78.676645147550	65.530816133675
C	126.190421617600	79.088845828851	60.480689616768
O	125.173123913629	77.182296448738	55.131229864509
C	121.556424504628	74.205334752053	58.240229629057
C	119.210166030696	75.471530080337	63.800419130921
C	123.892268430401	79.253295120178	65.009604432388
C	125.949040816220	78.275235094613	59.358853722486
C	121.464034925240	74.842014244412	59.618675525809
C	120.372212199173	76.341885698042	63.586217864652
C	123.935409910068	78.884955016323	63.601844147221
C	124.777293683747	77.516151354295	59.662179739151
N	122.842971807808	78.101434497322	63.285868581319
Mg	122.406431329786	77.193622026610	61.437266430989

H	124.975161960351	73.750988778482	57.469476011079
H	123.648125699884	72.635282146572	57.143914862174
H	118.613074299862	76.317801735338	65.687105637463
H	124.511149453052	80.538954562648	66.594273954241
H	125.349149925690	80.816716456105	65.074999772525
H	117.257505301403	73.681026594656	64.849169561439
H	117.013440941041	74.695858456291	66.377596581494
H	126.884295054897	79.748435752088	66.714292271044
H	126.585327394149	78.614428736619	65.370525497249
H	125.760759391741	78.384126668556	66.925217541329
H	125.883463277266	75.956306882665	57.132569648985
H	125.058193074102	77.489313513302	53.114253547491
H	124.751636843290	78.964122939374	54.091610261402
H	123.500997416245	77.673248248610	53.969613138902
H	119.746376854333	73.766012697037	60.172187416832
H	120.435880276018	77.231038783223	65.507455054341
H	125.705631751763	79.941232796863	63.100965657609
H	120.531930371888	74.274810317415	56.317592670963
H	120.555112581612	75.832005902459	57.183199709598
H	119.465119949581	74.539626887441	57.722292416005
H	117.088127773630	73.910963057089	62.683377231249
H	118.332807778256	72.680027436565	62.916976304528
H	118.070658622018	73.368820801586	61.307560047853
H	122.697304016281	79.574783156713	67.480504196769
H	122.610157099642	77.801308070430	67.488929813175
H	121.190149220265	78.754689038351	67.018779304501
H	127.301536218296	80.464758517671	61.707439622670
H	127.005570169873	81.008583058462	60.040581450452
H	128.231740280063	79.755071601844	60.363337561589
H	122.973738053689	74.975440161005	56.755636948486
H	121.444083669951	73.114212204858	58.311906512437
H	122.764130245710	84.724519649378	60.515645065281
H	119.464784702153	82.394796200562	59.293341891174
H	124.146738270111	72.958577772272	58.816146234436

Chlz-D1

85

C	74.262563985213	71.123412331221	80.117565905605
H	73.769119465405	71.791709448703	80.836845934254
H	73.714470602571	71.175474918901	79.176104413151
C	74.245771185811	69.719040797239	80.602696086463
N	74.330675436432	69.393259259388	81.939210128041
H	74.346849822224	70.084876872216	82.695319247842
C	74.267613373371	68.051122322530	82.072028949681
H	74.352929362359	67.551618907842	83.032252395283
N	74.156662561833	67.480554495341	80.873713375949
C	74.142889422558	68.508559140395	79.952877337883
H	74.059758776347	68.303176523821	78.891229153148
C	78.530702283773	63.997715282684	83.267952862898
C	75.462887735030	64.992402060849	75.101818173794
C	69.072378397676	63.466921866207	79.628329005121
C	74.136383715966	65.007163678305	85.843619663373
N	76.343713104462	65.441916625541	81.580743439550
C	76.215830422429	65.323990283855	74.036047866856
C	68.981431869899	61.958042756539	79.910291326950
C	75.600705893942	65.424580050728	85.355773709265
N	75.370184029827	65.272846447487	78.772471341957

O	73.788087442666	64.947012566673	87.012620579339
C	75.817032200061	66.896056808054	85.720460402597
N	73.590117203864	64.737501838497	82.350465250437
C	76.470937078026	68.408251756296	87.419707758570
C	75.566425203347	65.235457824859	83.848415272745
C	77.625700435104	65.888572978265	79.542443249160
C	73.446857385197	64.632725016687	77.381933812665
C	71.293718097776	64.203958518299	81.716813946557
C	78.508840004928	67.746761079346	82.131294292813
C	78.294764638389	65.737011148012	76.479290614429
C	70.488067949114	63.780360054283	76.753806725635
C	70.860645256101	64.124669151139	84.870909567382
C	76.587750503446	65.408635285805	82.927749945478
C	76.683190944007	65.587759875900	78.537340655600
C	72.456875258925	64.431133062245	78.359419429843
C	72.276338600034	64.438782821271	82.687721545074
O	75.501259095278	67.823957192466	84.989943810698
C	78.089832385185	65.472698994723	83.180172525315
C	76.963855880532	65.522173088187	77.117668326017
C	71.084187432652	64.023365853607	78.101688033781
C	72.114451330334	64.426899331243	84.129230241359
O	76.355782261258	67.038312483467	86.933487447745
C	78.550577219300	66.223270120306	81.914447347436
C	75.768838978915	65.166569507628	76.499109412164
C	70.469675107899	63.913152183320	79.332046954175
C	73.373387095561	64.758923759988	84.633742750516
C	77.471695246275	65.813849516401	80.923608863968
C	74.783520338753	65.004051459232	77.563012748887
C	71.480475596459	64.244573147007	80.327035069430
C	74.231601597352	64.929845967609	83.507586401159
N	72.662123975633	64.570846100616	79.702123653288
Mg	74.398075308809	65.381932494002	80.584829363763
H	78.123374935534	63.443671491203	82.409653732868
H	78.099344961254	63.559463665919	84.175996547512
H	74.481659229878	64.556889258656	74.891596990943
H	68.412666414429	63.721557931165	78.783621761563
H	68.674967460098	64.023265837510	80.492292713886
H	77.200574423621	65.773380600454	74.135033527194
H	75.827556880915	65.163513943208	73.031714308286
H	69.614955586010	61.682014235543	80.765669745253
H	69.328622084370	61.378312760959	79.042329372907
H	67.947979397865	61.656204171961	80.135350490374
H	76.333293270414	64.798092059634	85.881170519182
H	75.471882399834	68.848488306235	87.500109029877
H	77.073223454263	69.007058270201	86.729593778516
H	76.949748108558	68.338612794682	88.399520299366
H	78.616930010492	66.190687693350	79.200156961548
H	73.136633717054	64.439660541745	76.355246295932
H	70.292788346103	63.953817049091	82.072773203721
H	79.228226274248	68.045136842923	82.907563393426
H	77.506267891932	68.061682710944	82.458449321710
H	78.753113873194	68.287876616527	81.206641825600
H	78.521507879540	64.934016387063	75.760043581878
H	79.103835145693	65.761679041390	77.220300261128
H	78.332034648803	66.685749186380	75.919336492213
H	70.721367481134	64.608323395998	76.067789316412
H	69.395202025092	63.680856478685	76.812014064459
H	70.873382445384	62.862433644399	76.281155295019

H	70.315071681284	63.289352547383	84.408877010274
H	70.173489935444	64.984990433343	84.882180359927
H	71.089585874695	63.871466727396	85.911884344828
H	78.357156858166	66.013662744120	84.098746856976
H	79.551459072961	65.925325060334	81.573082536503
H	75.289165037607	71.507529442906	79.946925804049
H	79.616943226798	63.779397450177	83.304050133430

REFERENCES

1. Y. Umena, K. Kawakami, J.-R. Shen and N. Kamiya, *Nature*, 2011, **473**, 55-60.
2. S. Schott-Verdugo and H. Gohlke, *J. Chem. Inf. Model.*, 2019, **59**, 2522-2528.
3. J. A. Maier, C. Martinez, K. Kasavajhala, L. Wickstrom, K. E. Hauser and C. Simmerling, *J. Chem. Theory Comput.*, 2015, **11**, 3696-3713.
4. W. L. Jorgensen, J. Chandrasekhar, J. D. Madura, R. W. Impey and M. L. Klein, *J. Chem. Phys.*, 1983, **79**, 926-935.
5. C. J. Dickson, B. D. Madej, Å. A. Skjevik, R. M. Betz, K. Teigen, I. R. Gould and R. C. Walker, *J. Chem. Theory Comput.*, 2014, **10**, 865-879.
6. J. Wang, R. M. Wolf, J. W. Caldwell, P. A. Kollman and D. A. Case, *J. Comput. Chem.*, 2004, **25**, 1157-1174.
7. A. Sirohiwal and D. A. Pantazis, *Acc. Chem. Res.*, 2023, **56**, 2921-2932.
8. N. Sakashita, H. C. Watanabe, T. Ikeda, K. Saito and H. Ishikita, *Biochemistry*, 2017, **56**, 3049-3057.
9. F. Guerra, M. Siemers, C. Mielack and A.-N. Bondar, *J. Phys. Chem. B*, 2018, **122**, 4625-4641.
10. M. Ceccarelli, P. Procacci and M. Marchi, *J. Comput. Chem.*, 2003, **24**, 129-142.
11. F. Guerra, S. Adam and A.-N. Bondar, *J. Mol. Graphics Modell.*, 2015, **58**, 30-39.
12. P. Li and K. M. Merz, *Chem. Rev.*, 2017, **117**, 1564-1686.
13. I. S. Joung and T. E. Cheatham, III, *J. Phys. Chem. B*, 2009, **113**, 13279-13290.
14. I. S. Joung and T. E. Cheatham, III, *J. Phys. Chem. B*, 2008, **112**, 9020-9041.
15. R. J. Loncharich, B. R. Brooks and R. W. Pastor, *Biopolymers*, 1992, **32**, 523-535.
16. H. J. C. Berendsen, J. P. M. Postma, W. F. van Gunsteren, A. DiNola and J. R. Haak, *J. Chem. Phys.*, 1984, **81**, 3684-3690.
17. J.-P. Ryckaert, G. Ciccotti and H. J. C. Berendsen, *J. Comput. Phys.*, 1977, **23**, 327-341.
18. U. Essmann, L. Perera, M. L. Berkowitz, T. Darden, H. Lee and L. G. Pedersen, *J. Chem. Phys.*, 1995, **103**, 8577-8593.
19. D. A. Case, T. E. Cheatham III, T. Darden, H. Gohlke, R. Luo, K. M. Merz Jr., A. Onufriev, C. Simmerling, B. Wang and R. J. Woods, *J. Comput. Chem.*, 2005, **26**, 1668-1688.
20. R. Salomon-Ferrer, A. W. Götz, D. Poole, S. Le Grand and R. C. Walker, *J. Chem. Theory Comput.*, 2013, **9**, 3878-3888.
21. S. Barazzouk, L. Bekalé and S. Hotchandani, *J. Mater. Chem.*, 2012, **22**, 25316-25324.
22. F. Müh, M. Plöckinger, H. Ortmayer, M. Schmidt am Busch, D. Lindorfer, J. Adolphs and T. Renger, *J. Photochem. Photobiol., B*, 2015, **152**, 286-300.
23. Y. Shibata, S. Nishi, K. Kawakami, J.-R. Shen and T. Renger, *J. Am. Chem. Soc.*, 2013, **135**, 6903-6914.
24. P. Sarngadharan, S. Maity and U. Kleinekathöfer, *J. Chem. Phys.*, 2022, **156**.
25. K. Saito, K. Mitsuhashi, H. Tamura and H. Ishikita, *Biophys. J.*, 2023, **122**, 470-483.
26. F. H. M. Koua, Y. Umena, K. Kawakami and J.-R. Shen, *Proc. Natl. Acad. Sci. U. S. A.*, 2013, **110**, 3889-3894.
27. I. Schelter, J. M. Foerster, A. T. Gardiner, A. W. Roszak, R. J. Cogdell, G. M. Ullmann, T. B. de Queiroz and S. Kümmel, *J. Chem. Phys.*, 2019, **151**.
28. T. Yanai, D. P. Tew and N. C. Handy, *Chem. Phys. Lett.*, 2004, **393**, 51-57.
29. Y. Tawada, T. Tsuneda, S. Yanagisawa, T. Yanai and K. Hirao, *J. Chem. Phys.*, 2004, **120**, 8425-8433.
30. J.-D. Chai and M. Head-Gordon, *J. Chem. Phys.*, 2008, **128**.

31. N. Mardirossian and M. Head-Gordon, *Phys. Chem. Chem. Phys.*, 2014, **16**, 9904.
32. M. Casanova-Páez, M. B. Dardis and L. Goerigk, *J. Chem. Theory Comput.*, 2019, **15**, 4735-4744.
33. H. Iikura, T. Tsuneda, T. Yanai and K. Hirao, *J. Chem. Phys.*, 2001, **115**, 3540-3544.
